# Robust Porous WC‐Based Self‐Supported Ceramic Electrodes for High Current Density Hydrogen Evolution Reaction

**DOI:** 10.1002/advs.202106029

**Published:** 2022-03-25

**Authors:** Feihong Wang, Yutong Wu, Binbin Dong, Kai Lv, Yangyang Shi, Nianwang Ke, Luyuan Hao, Liangjun Yin, Yu Bai, Xin Xu, Yuxi Xian, Simeon Agathopoulos

**Affiliations:** ^1^ CAS Key Laboratory of Materials for Energy Conversion Department of Materials Science and Engineering University of Science and Technology of China Hefei Anhui 230026 P. R. China; ^2^ School of Materials Science and Engineering Henan Key Laboratory of Special Protective Materials Luoyang Institute of Science and Technology Luoyang Henan 471023 P. R. China; ^3^ School of Energy Science and Engineering University of Electronic Science and Technology of China 2006 Xiyuan Road Chengdu PR China; ^4^ School of Engineering Science University of Science and Technology of China Hefei Anhui 230026 P. R. China; ^5^ CAS Key Laboratory of Mechanical Behaviors and Design of Materials Department of Modern Mechanics University of Science and Technology of China Hefei Anhui 230026 P. R. China; ^6^ Department of Materials Science and Engineering University of Ioannina Ioannina GR‐451 10 Greece

**Keywords:** electrocatalyst, hydrogen evolution reaction, nitrogen‐doped WC/W heterostructure, porous ceramic membrane, self‐supported electrode

## Abstract

Developing an economical, durable, and efficient electrode that performs well at high current densities and is capable of satisfying large‐scale electrochemical hydrogen production is highly demanded. A self‐supported electrocatalytic “Pt‐like” WC porous electrode with open finger‐like holes is produced through industrial processes, and a tightly bonded nitrogen‐doped WC/W (WC‐N/W) heterostructure is formed in situ on the WC grains. The obtained WC‐N/W electrode manifests excellent durability and stability under multi‐step current density in the range of 30–1000 mA cm^−2^ for more than 220 h in both acidic and alkaline media. Although WC is three orders of magnitude cheaper than Pt, the produced electrode demonstrates comparable hydrogen evolution reaction performance to the Pt electrode at high current density. Density functional theory calculations attribute its superior performance to the electrode structure and the modulated electronic structure at the WC‐N/W interface.

## Graphical abstract

Well established industrial processes are employed to produce self‐supported electrocatalytic WC porous electrode, which features open finger‐like holes and tightly bonded nitrogen‐doped WC/W heterostructure. The electrode manifests excellent stability under multi‐step current density in the range of 30–1000 mA cm^−2^ for more than 220 h in acidic and alkaline media.

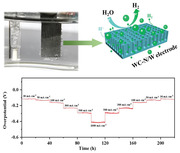

## 1 Introduction

In the light of the global energy crisis related to the diminishing resources of traditional fossil fuels, hydrogen is a promising alternative, clean, and sustainable energy source.^[^ ^1^ ^]^ Among various hydrogen production schemes, electrocatalytic water splitting manifests inherent advantages, such as high product purity, large‐scale production feasibility, and environmentally friendly technology.^[^ ^2^ ^]^ Pt‐based metal catalysts are the most effective electrocatalysts for hydrogen evolution reaction (HER). Nonetheless, their high cost and the limited natural resources severely hinder their large‐scale application.^[^ ^3^ ^]^ Accordingly, tremendous efforts are devoted to developing cost‐effective and highly efficient advanced non‐noble metallic electrochemical catalysts.^[^ ^4^ ^]^

Transition metal carbides (TMC), such as WC,^[^ ^5^ ^]^ Mo_2_C,^[^ ^6^ ^]^ Ni_3_C,^[^ ^7^ ^]^ etc., have been considered as promising electrocatalysts to replace noble metals due to their abundant earth reserves and excellent electrical conductivity. For instance, tungsten carbide (WC) is a promising candidate for transition metal‐based HER electrocatalysts due to the low cost, the Pt‐like electronic properties, and the stable catalytic performance.^[^ ^1^, ^5^, ^8^ ^]^ Nevertheless, typical WC catalyst synthesis methods, such as carbonization of tungsten (W) or W‐based precursors, result in bulk materials or nanoparticles with poor crystallinity and impurities, which negatively affect HER performance. Moreover, the intermediate product will also cause pollution and create harsh experimental conditions.^[^ ^3^, ^9^ ^]^ Furthermore, in the course of the electrochemical test, the WC powder needs to be coated on the conductive electrodes with the aid of poorly conductive polymeric binders, which cover many active sites. Therefore, the charge transfer is delayed. In addition, the coated catalyst is also easily peeled off from the substrate during long‐term or high‐current operation due to the weak adhesion between the catalyst and the electrode.^[^ ^10^ ^]^

To achieve large‐scale electrochemical hydrogen production, an economical, durable, and efficient electrode with a controllable porous structure that works well at high current densities must be used.^[^ ^11^ ^]^ Porous conductive ceramics are attractive candidates for self‐supported electrodes because of their high thermal and chemical stability, easy tailoring of pore structure, and high mechanical strength.^[^ ^12^ ^]^ Additionally, the inherent hydrophilic properties of ceramic surfaces enhance the contact between the catalyst and the electrolyte, facilitate the transfer of charge and ions, and favor the detachment of bubbles.^[^ ^13^ ^]^

This paper reports on the successful preparation of a WC porous ceramic membrane through a phase‐inversion tape‐casting and pressureless sintering method. This well‐established, efficient, and economical forming method can accurately customize the membrane thickness. Thus, it is suitable for large‐scale industrial production. This study aims to obtain the demanded characteristics, briefly outlined as follows, along with several key features of the production process. First of all, the produced ceramic membranes must contain oriented finger‐like holes,^[^ ^14^ ^]^ which can increase the effective area of the electrode. Moreover, lower overpotentials in acidic and alkaline media than those of the metallic or carbon substrates should be achieved.^[^ ^15^ ^]^ The HER performance will be expected to be improved through the in situ grain surface modification by heat treatment under NH_3_ atmosphere, whereby a nitrogen‐doped WC/W (WC‐N/W) heterostructure will be formed at the surface of the WC grains. The stable atomic conjugation of the active surface can favor the rapid charge transfer and improve long‐term stability,^[^ ^16^ ^]^ especially at high current density. Density functional theory (DFT) computations were performed to shed light on the good HER performance of WC‐N/W heterostructure.

## 2 Results and Discussion

### 2.1 Structural Features

**Figure 1:**
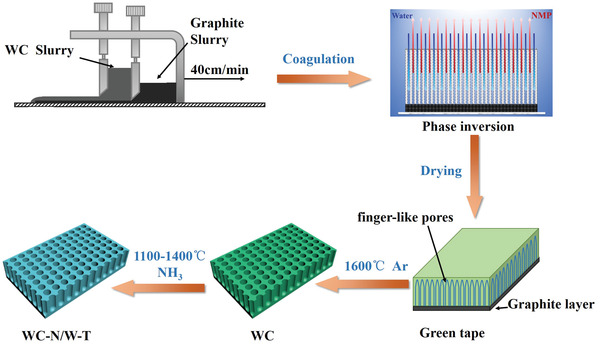
The preparation procedure of the self‐supported porous WC‐based electrodes.

The details of the production of the WC‐N/W electrode are described in Section 4. However, to help the reader understand better the results presented in the following paragraphs, the fundamental steps of the preparation process are outlined here. In brief, the WC‐N/W electrode was prepared by a simple combination of phase‐inversion tape‐casting and pressureless sintering method, as depicted in **Figure** **1**.^[^ ^17^ ^]^ According to the typical process, WC and graphite slurries were initially prepared, whose compositions are listed in Table S1, Supporting Information. Then, two‐layer tapes were obtained by tape‐casting, which were immersed afterward in a water bath where they were immediately solidified. Next, NMP (*N*‐methyl‐2‐pyrrolidone) and water were diffused to form an unstable phase system. This allows phase inversion to occur, resulting in the formation of a three‐layered structure, comprising a skin layer, a finger‐like pore layer, and a sponge layer. Finally, the green membranes were sintered in Ar at 1600 °C, followed by thermal treatment under NH_3_ atmosphere at 1100–1400 °C to obtain the final WC‐N/W heterostructure on the WC grain surface. The products are denoted as WC‐N/W‐*T* (where *T* represents the thermal‐treatment temperature).

**Figure 2:**
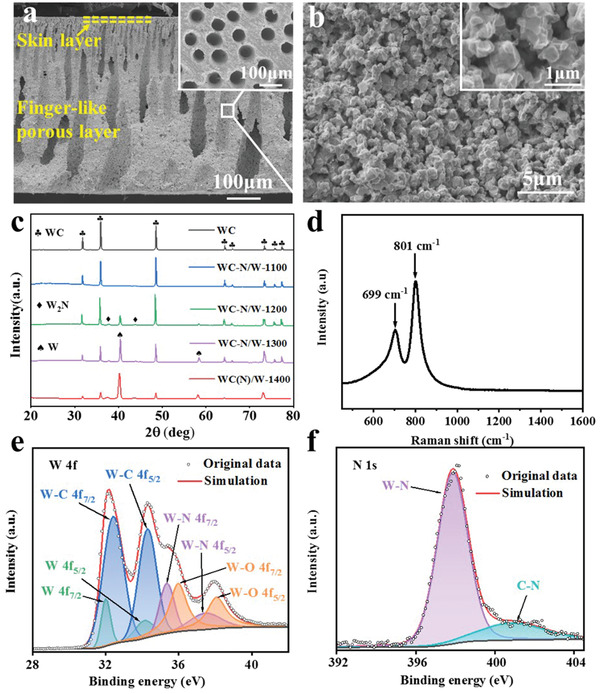
Microstructure of WC‐N/W‐1200 electrode as seen in a) an overall cross‐section SEM image (the inset illustrates the bottom surface) and b) the morphology inside the membrane (the inset shows a magnified view). c) X‐ray diffractograms of the WC based electrodes green sample and heat‐treated at various temperatures. d) Raman spectrum and high‐resolution XPS spectrum of e) W 4f and f) N 1s of the WC‐N/W‐1200 electrode.

The typical morphology of the cross‐section of the WC‐N/W‐1200 electrode is shown in **Figure** **2**a. The membrane with a thickness of ≈0.6 mm retained the asymmetric porous structure with skin and finger‐like pore layers. The upper skin layer was relatively dense, with a thickness of about 20 µm. The lower layer contained larger finger‐like holes with obvious vertical orientation directed toward the bulk from the skin layer. The graphite in the sponge layer disappeared (Figure S1a,b, Supporting Information). Large open holes with an average diameter of ≈60 µm were uniformly distributed at the bottom surface (inset of Figure 2a), allowing the entrance of electrolyte and favoring gas discharge during the electrolysis process. The internal skeleton and the surface of the skin layer of the ceramic membrane were porous (64.2% porosity) with interconnected grains (inset of Figure 2b; Figure S2, Supporting Information). High flexural strength of 77.2 MPa was measured. There is a negligible influence of thermal treatment under NH_3_ at various temperatures on the microstructure of WC‐N/W electrodes (Figure S1c,d, Supporting Information). Compared to nanostructured surfaces constructed by nanosheets or nanowires,^[^ ^18^ ^]^ the smoothness and compactness of the grain surface might be unfavorable for electrocatalytic performance. Still, it can improve the long‐term stability at high current density. The pore size distribution of the membrane was very narrow, with an average pore size of 0.59 µm (Figure S3a, Supporting Information). Brunauer–Emmet–Teller (BET) analysis (Figure S3b, Supporting Information) showed that WC‐N/W‐1200 had a low specific surface area of 0.750 m^2^ g^−1^, and a low size distribution in the mesopore region of 0.0024 cm^3^ g^−1^, which is in good agreement with scanning electron microscopy (SEM) observations.

According to the X‐ray diffractogram of the WC‐N/W electrode heat‐treated at 1100 °C (Figure 2c), WC was exclusively identified (JCPDS No. 51–0939). With the increase of temperature, formation of traces of W_2_N can be suggested, due to the newly formed diffraction peaks at 37.73° and 43.85° corresponding to the (111) and (200) planes of this phase (JCPDS No. 25–1257).^[^ ^19^ ^]^ The presence of metallic W (JCPDS No. 04–0806) was recorded at 1200 °C, whose content increases with increasing temperature; thereby, the grain surface should be almost totally covered by metallic W at 1400 °C.

Accordingly, the following mechanism is proposed for the formation of WC‐N/W heterostructure. First, NH_3_ is adsorbed on the surface of WC, protons are removed, and then N enters the WC lattice and gradually replaces C to form W_2_N, according to the reaction:^[^ ^20^ ^]^

(1) $$4\mathrm{WC}+6NH_{3}\to 2W_{2}N+4\mathrm{HCN}+7H_{2}$$

Since W_2_N is unstable at high temperatures, it decomposes into W and N_2_:^[^ ^21^ ^]^

(2) $$2W_{2}N\;\to 4W+N_{2}↑$$

Raman spectrum (Figure 2d) casts light on WC‐N/W‐1200 electrode composition. The characteristic peaks at around 699 and 801 cm^−1^ are assigned to the W—C and W—N stretching modes.^[^ ^3^, ^4^, ^19^, ^22^ ^]^ X‐ray photoelectron spectroscopy (XPS) determined the chemical environment of each element in the WC‐N/W‐1200 membrane. The XPS full‐spectrum (Figure S4, Supporting Information) shows the presence of the four elements of W, C, O, and N in the membrane. The W 4f spectral line can be deconvoluted into eight peaks (Figure 2e), which are attributed to the metallic tungsten (31.6 and 33.8 eV),^[^ ^23^ ^]^ W—C bonds (32.4 and 34.3 eV),^[^ ^2^, ^24^ ^]^ W—N bonds (35.3 and 37.6 eV),^[^ ^19^, ^22^ ^]^ and W—O bonds (36.0 and 38.1 eV). The W—O bonds should result from the inevitable WC surface oxidation.^[^ ^2^, ^23^, ^25^ ^]^ Due to the tiny amount of W_2_N, it is proposed that the strong peaks of W—N bonds are ascribed to the nitrogen incorporated into the WC crystals, suggesting the gradual removal of carbon by nitrogen.

The W 4f high‐resolution XPS spectra of the other electrodes are shown in Figure S5, Supporting Information. Only W—O and W—C bonds were identified in the WC and the WC‐N/W‐1100 electrodes. The peaks of W—N bonds and metallic W were detected when the thermal‐treatment temperature was higher than 1100 °C, which is consistent with the X‐ray diffraction (XRD) results. The peaks at 397.8 and 400.8 eV in the N 1s spectrum of WC‐N/W‐1200 (Figure 2f) clearly confirm the W—N and C—N bonds, respectively.^[^ ^19^, ^22^, ^26^ ^]^

**Figure 3:**
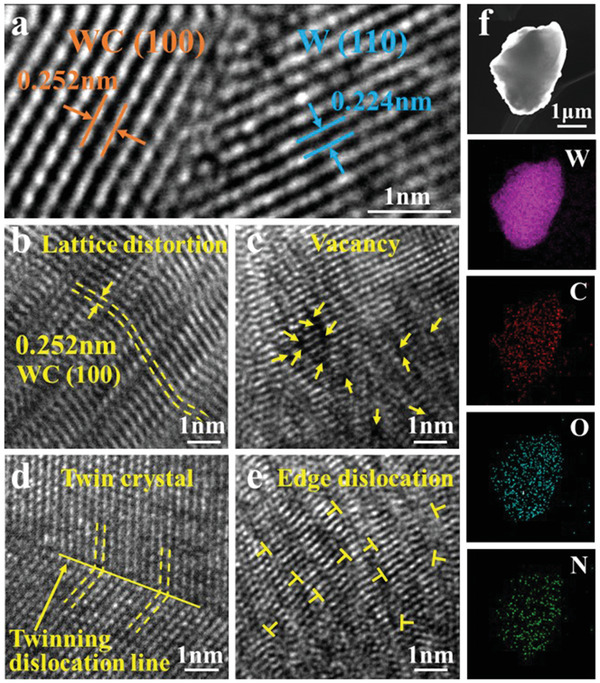
HRTEM images, which show a) the heterostructure and b–e) various types of defects in the WC‐N/W‐1200 electrode, and f) element distribution (by TEM‐EDS analysis).

The high resolution transmission electron microscopy (HRTEM) of the WC‐N/W‐1200 electrode (**Figure** **3**a) suggests that the WC‐N/W heterostructure was constructed with an obvious interface. The lattice fringes of 0.252 and 0.224 nm are assigned to the (100) plane of the hexagonal WC and the (110) plane of the cubic metal W, respectively. The work functions of WC (100) and W(110) facets are calculated as 5.11 and 3.99 eV, respectively, which means that WC accepts electrons from W particles until the Fermi level reaches equilibrium. The results of XPS analysis reveal the redistribution of electrons on the WC‐N/W interface (Figure S6, supporting information). The binding energy of the characteristic W 4f XPS peak of W in WC‐N/W‐1200 is higher than that of single‐phase W. This is an indication of electrons transfer from W to WC in WC‐N/W heterostructure, which is a reflection of the Mott–Schottky effect.^[^ ^27^ ^]^ The higher and wider binding energy of W‐C peak in WC‐N/W‐1200, compared to that in WC single‐phase, is attributed to the doping effect of the strongly electronegative N element, ^[^ ^28^ ^]^ which is consistent with the previous XPS analysis results. And also, in the N‐free WC/W heterostructure, the binding energy of the W peak is higher than that of the single‐phase W, and the binding energy of the W‐C peak is lower than that of the single‐phase WC, which also indicates that electrons transfer from W to WC at the WC/W interface. Moreover, the positive slope of the Mott–Schottky plots for the WC‐N/W‐1200 sample indicates the *n*‐type nature (Figure S7, Supporting Information).^[^ ^29^ ^]^ The resultant Schottky barrier will obviously cause the redistribution of electrons at the interface between W and WC, and gradually enrich the positive charge on the metal W side.^[^ ^1^, ^5^, ^30^ ^]^ At high temperatures, a large amount of N atoms should be doped into the WC lattice, resulting in the formation of various types of defects, such as lattice distortion, vacancies, twin‐crystal, and edge dislocation structures, as shown in Figure 3b–e, respectively.^[^ ^31^ ^]^ The existence of a large number of anion vacancies was also confirmed by electron paramagnetic resonance (EPR) spectroscopy, where the WC had a very weak signal, while a strong resonance line appeared at *g* of 2.0026 in the WC‐N/W‐1200 electrode (Figure S8, Supporting Information).^[^ ^32^ ^]^ These defects can effectively alter the periodic structure of the crystal and affect the chemical properties and the electronic structure of the catalyst, improving the intrinsic catalytic activity and exposing more active sites, thereby the HER performance is improved.^[^ ^33^ ^]^ These findings confirm that NH_3_ gradually destroyed the surface crystal lattice of WC grains, and N was doped into the WC crystal lattice, which is in good agreement with the XPS and Raman spectra.^[^ ^31^, ^34^ ^]^

Transmission electron microscope coupled energy dispersive spectroscopy (TEM‐EDS) elemental analysis suggests that W, C, and O were uniformly distributed in the WC‐N/W‐1200 electrode (Figure 3f). Due to the presence of the O element, it is proposed that the internal WO_3_ in the WC green body (Table S1, Supporting Information) was transformed into an amorphous glass‐phase when it was sintered at 1600 °C, and inevitably caused surface oxidation.^[^ ^23^, ^35^ ^]^ The distribution of the N element is not as uniform as the other elements, revealing that a part of WC was doped with N.

### 2.2 HER Performance

**Figure 4:**
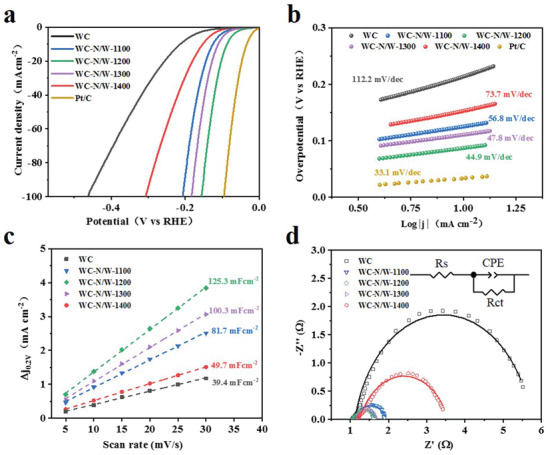
HER performance of WC and WC‐N/W electrodes heat‐treated at various temperatures in 0.5 m H_2_SO_4_. a) Polarization curves and b) the corresponding Tafel plots. c) *C*_dl_ at a potential of 0.2 V (vs. RHE). d) EIS Nyquist plots (the inset shows the equivalent circuit).

The electrocatalytic HER activity was measured using a standard three‐electrode device in acidic and alkaline media (Figure S9, Supporting Information). The HER performance of Pt/C, WC, and WC‐N/W electrodes in 0.5 m H_2_SO_4_ solution is demonstrated in **Figure** **4** and Table S2, Supporting Information. The polarization curve after *i*–*R* compensation (Figure 4a) shows that the Pt/C catalyst exhibited the lowest overpotential (current density of 10 mA cm^−2^) of 34 mV. WC had the highest overpotential of 213 mV among the five WC electrodes, which is lower than many metallic substrates.^[^ ^15^, ^36^ ^]^ With the increase of the thermal‐treatment temperature, the HER activity was enhanced and then weakened. Hence, WC‐N/W‐1200 showed the best HER activity with an overpotential of 87 mV.

The Tafel plots in Figure 4b, obtained by fitting the polarization curves with the Tafel equation, were employed to investigate the kinetics of the HER reaction process. The Tafel slope of Pt/C is 33.1 mV dec^−1^, displaying the fastest HER reaction kinetics, consistent with literature.^[^ ^37^ ^]^ On the other hand, the Tafel slope of WC‐N/W‐1200 is 44.9 mV dec^−1^, which is lower than WC, WC‐N/W‐1100, WC‐N/W‐1300, and WC‐N/W‐1400 (112.2, 56.8, 47.8, and 73.7 mV dec^−1^, respectively).

In acidic media, the mechanism of the HER process can be elucidated in the following three steps:^[^ ^4^, ^37^ ^]^

(3) $$\mathrm{Volmer}\;\mathrm{reaction}\;,H_{3}O^{+}+e^{-}\to H_{\mathrm{ads}}+H_{2}O$$

(4) $$\mathrm{Heyrovsky}\;\mathrm{reaction}\;H_{3}O^{+}+e^{-}+H_{\mathrm{ads}}\to H_{2}↑+H_{2}O$$

(5) $$\mathrm{Tafel}\;\mathrm{reaction}\;H_{\mathrm{ads}}+H_{\mathrm{ads}}\to H_{2}↑$$

Accordingly, the lowest Tafel slope of WC‐N/W‐1200 reveals that the Volmer–Heyrovsky mechanism governs its HER kinetics in H_2_SO_4_. In addition, the overpotential of the WC/W electrode is 102 mV, and the Tafel slope is 49.8 mV dec^−1^, which are both worse than WC‐N/W‐1200, suggesting that N doping is beneficial to improve the HER performance (Figures S10 and S11, Supporting Information).

The exchange current density (*j*_0_), which is another crucial kinetics parameter for evaluating HER activity, was calculated from the cathodic current density of the Tafel equation at zero overpotential. From Figure S12 and Table S2, Supporting Information, the value of *j*_0_ of WC‐N/W‐1200 was determined as 0.192 mA cm^−2^, which is superior to the other electrodes prepared, except Pt/C, which demonstrates the highest catalytic efficiency and HER activity. The electrochemically active surface area (ECSA) of the electrocatalysts can be obtained by the double‐layer capacitance (*C*_dl_), acquired by the cyclic voltammetry (CV) measurement,^[^ ^38^ ^]^ as shown in Figure 4c and Figure S13, Supporting Information. The *C*_dl_ of WC‐N/W‐1200 was 125.3 mF cm^−2^, which is markedly higher than WC, WC‐N/W‐1100, WC‐N/W‐1300, and WC‐N/W‐1400 (39.4, 81.7, 49.7, and 100.3 mF cm^−2^, respectively), suggesting that this electrode exhibits the highest number of exposed electrochemically active sites. It is worth noting that, owing to the open pore structure of the membrane, it is proposed that *C*_dl_ can be further significantly improved by optimizing the thermal‐treatment procedure.^[^ ^12a^ ^]^ Moreover, the intrinsic activity of the catalyst can be evaluated by calculating the ECSA. The WC‐N/W‐1200 electrode exhibited the largest ECSA among the prepared membrane electrodes in 0.5 m H_2_SO_4_ (Table S3, Supporting Information).^[^ ^39^ ^]^ Furthermore, according to the linear sweep voltammetry (LSV) curves normalized by ECSA, the WC‐N/W‐1200 electrode still showed the highest catalytic performance, demonstrating the best intrinsic activity (Figure S14, Supporting Information). Electrochemical impedance spectroscopy (EIS) (Figure 4d) was employed further to investigate the catalytic kinetics of the HER process. The semicircle in the Nyquist plots represents the charge transfer process at the interface of the electrocatalyst and the electrolyte, reflecting the magnitude of the charge transfer resistance (*R*_ct_, Table S2, Supporting Information). WC‐N/W‐1200 had the smallest radius, suggesting that it had the lowest *R*_ct_ (0.557 Ω). This finding indicates its fast charge transfer rate and HER kinetics. The Faraday efficiency (FE) of the membrane electrode was evaluated, and the agreement between the experimentally measured and the theoretically calculated amount of hydrogen (assuming 100% FE) suggests that the Faraday efficiency is close to 100% (Figure S15, Supporting Information).

**Figure 5:**
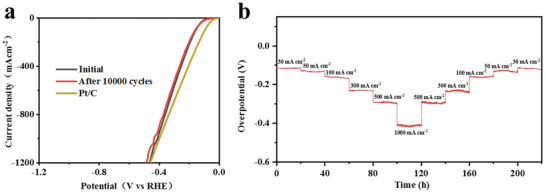
HER performance and results of stability test of WC‐N/W‐1200 electrode in 0.5 m H_2_SO_4_. a) Polarization curves before and after 10 000 CV cycles for durability test and b) chronopotentiometry curve at 30, 50, 100, 300, 500, and 1000 mA cm^−2^ for 220 h.

The durability of the WC‐N/W‐1200 electrode was evaluated by comparing the polarization curves before and after 10 000 CV cycles. As shown in **Figure** **5**a, the two polarization curves are almost overlapped. It is also worth mentioning that it was close to Pt/C at high current densities.^[^ ^11b,c^ ^]^ Moreover, the microstructure, chemical states, and phase composition were not changed (Figures S16–S19, Supporting Information). Hence, these results indicate the excellent durability in acidic media. Furthermore, according to the chronopotentiometry curve test at 30, 50, 100, 300, 500, and 1000 mA cm^−2^ (Figure 5b), the overpotential remained almost constant under various tested current densities. After 220 h of operation, the overpotential at 30 mA cm^−2^ remained almost constant, demonstrating excellent long‐term stability. Taking into account the scouring and impact effects of water flow on the electrode during the actual application of electrolyzed water, the water permeability test was carried out on the WC‐N/W‐1200 electrode. The water permeation flux was 14.26 × 10^3^ L m^−2^ h^−1^ bar^−1^, and remained stable under 3.0 bar for 4 h, suggesting excellent mechanical performance. Moreover, the LSV curves before and after the test almost overlap, suggesting practical and stable performance (Figure S20, Supporting Information).

**Figure 6:**
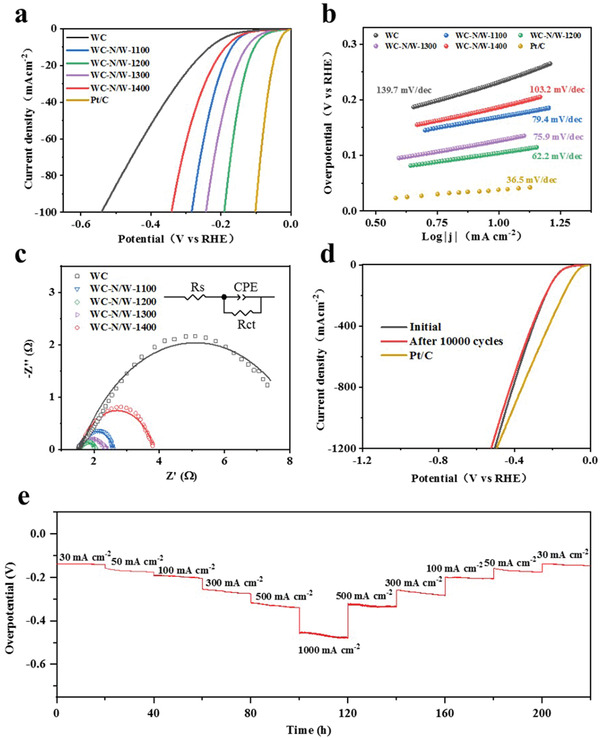
HER performance of WC and WC‐N/W electrodes in 1 m KOH. a) Polarization curves and b) the corresponding Tafel plots. c) EIS Nyquist plots (the inset shows the equivalent circuit). d) Polarization curves of WC‐N/W‐1200 electrode before and after 10 000 CV cycles. e) Chronopotentiometry curve at 30, 50, 100, 300, 500, and 1000 mA cm^−2^ for 220 h.

The HER electrocatalytic performance was also evaluated in 1 m KOH solution (**Figure** **6**). The electrode WC‐N/W‐1200 displayed the sharpest polarization curve slope, compared to the other WC‐based electrodes (Figure 6a; Table S4, Supporting Information), with an overpotential of 104 mV. Moreover, its Tafel slope is 62.2 mV dec^−1^, which is lower than that of other electrodes, which occurred through a Volmer–Heyrovsky route (Figure 6b). These performances were also better than WC/W electrode (the overpotential was 129 mV and the Tafel slope was 76.9 mV dec^−1^, Figure S21, Supporting Information). This electrode manifested the largest exchange current density *j*_0_ of 0.311 mA cm^−2^ and *C*_dl_ of 109.1 mF cm^−2^, revealing the fastest reaction kinetics and the most active area, respectively (Figures S22–S24 and Table S4, Supporting Information). Furthermore, according to the calculated ECSA and the LSV curves normalized by ECSA, the WC‐N/W‐1200 electrode demonstrated the highest catalytic performance and the best intrinsic activity (Figure S25 and Table S3, Supporting Information). The radius of the EIS curve of the WC‐N/W‐1200 showed the lowest charge transfer resistance of 0.514 Ω (Figure 6c; Table S4, Supporting Information). Moreover, Faraday efficiency was close to 100% (Figure S26, Supporting Information), suggesting that WC‐N/W‐1200 has high catalytic performance. As expected, 10 000 CV cycles had a negligible effect on the polarization curve, morphology, and phase composition of WC‐N/W‐1200 (Figure 6d; Figures S16–S19, Supporting Information). Moreover, after more than 200 h of operation, the overpotential remained nearly constant at 30 mA cm^−2^ (Figure 6e), reflecting its superior durability and stability in alkaline media.

At high current density, the rapidly produced hydrogen cannot be discharged from the pores in time, causing a block of the pores and, therefore, increasing the overpotential. This can be solved by increasing the finger pole diameter or improving the hydrophilicity of the electrode (Figure S27, Supporting Information).

Consequently, the thermal treatment under NH_3_ enhanced the catalytic activity of the WC‐based electrode, and the obtained WC‐N/W‐1200 demonstrated the best HER performance both in acidic and alkaline media. The HER performance of WC‐N/W‐1200 along with previously reported tungsten carbide‐based and metal carbide catalysts in acidic and alkaline media are outlined in Tables S5–S8, Supporting Information. The excellent performance and the simple and controllable preparation method qualify the WC‐N/W‐1200 as a competitive catalytic electrode material. In addition, the investigated system exhibits the following two important advantages. First, the prepared electrode displays excellent stability at high current intensity (to our knowledge, HER performance at high current intensity has never been reported). Second, the preparation process in this study is simple, cost‐effective, and suitable for large‐scale industrial hydrogen production.

### 2.3 DFT Calculations

**Figure 7:**
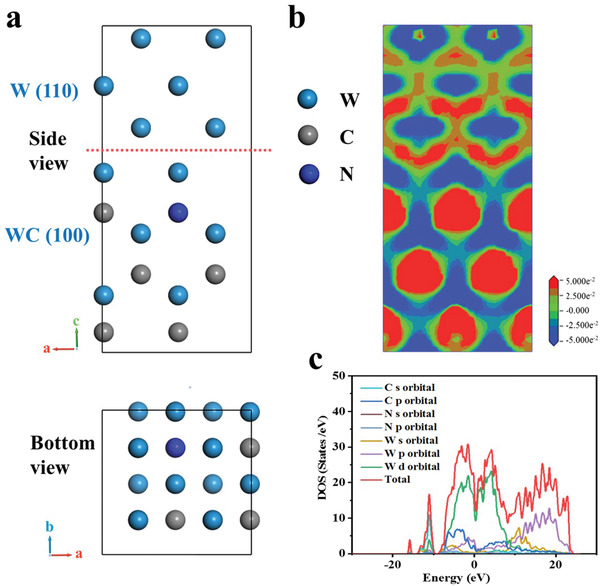
Results of DFT calculations conducted for determining the properties of WC‐N (100) /W (110) (N replaced 1/12 C) heterostructure. a) Schematic representation of the model of WC‐N/W heterostructure, b) slice of charge density difference at the WC‐N/W interface, and c) DOS of WC‐N/W heterostructure.

Density functional theory (DFT) calculations were performed to shed light on the relationship between the electronic structure and the superior electrocatalytic performance of WC/W heterostructure. According to the results of electron probe microanalyzer (EPMA) element analysis (Table S9, Supporting Information), the heterostructure was constructed by WC‐N (100) (N replaced 1/12 C) and W (110) (**Figure** **7**a). The calculated lattice parameters are tabulated in Table S10, Supporting Information. The charge density distribution at the WC‐N (100) /W (110) interface is illustrated in Figure 7b, where the red and blue colors represent the positron and negatron clouds, respectively. Obviously, the electron clouds are rearranged, and the interface accumulates more electron clouds, which should positively affect the catalytic activity of the heterostructure.

The partial density of states (PDOS) of the heterostructure is presented in Figure 7c. It is clearly observed that the 5d orbitals of W contribute most to the total density of state (TDOS) near the Fermi level, which suggests an enhanced carrier density. Meanwhile, the 2p orbitals of C and the 5p orbitals of W are overlapped, forming metallic bonds. The continuous TDOS near the Fermi level suggests that the WC‐N (100) /W (110) heterostructure exhibits metallic properties, which are much higher than the TDOS of WC (100) near the Fermi level (Figure S28, Supporting Information).

A model for WC (100) /W (110) was also established to study the differential charge density and density of state (DOS. The results are similar to those for WC‐N (100) /W (110) (Figures S29–S31, Supporting Information). It is worth mentioning that the TDOS near the Fermi level is slightly lower than WC‐N (100) /W (110), revealing that the doping of N enhances the charge transfer ability.

**Figure 8:**
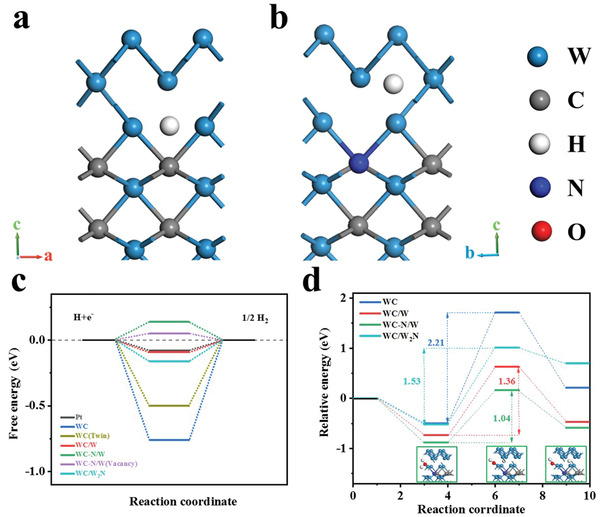
Optimized structure of adsorbed H on the a) WC (100) /W (110), and b) WC‐N (100) /W (110) (N replaced 1/12 C) heterostructure models. c) The calculated hydrogen adsorption energy diagram of the constructed models and Pt (111) surfaces. d) Free energy of each electrode model during water dissociation (insets show the structure of the catalyst at different stages).

The Gibbs free energy of hydrogen absorption was generally considered the most essential parameter to assess the catalysis performance of HER. Hence, a suitable catalyst should have a thermoneutral Δ*G*_H_ value (i.e., Δ*G*_H_ ≈ 0 eV). To evaluate the Δ*G*_H_ of the investigated heterostructures, the hydrogen adsorption on WC (100) /W (110) and WC‐N (100) /W (110) interfaces, as well as pure WC (100), were constructed (**Figure** **8**a,b; Figure S32, Supporting Information). As shown in Figure 8c, the Δ*G*_H_ of pure WC (100) is −0.76 eV, which is higher than that of Pt (111) that is −0.08 eV, suggesting that the catalytic activity of WC was weak.^[^ ^40^ ^]^ When the WC (100) /W (110) heterostructures are formed, the Δ*G*_H_ decreases to −0.09 eV, which suggests that the H atom adsorption in the heterostructure is thermodynamically favorable, and the heterointerface is an active center of HER. The value of Δ*G*_H_ of the WC‐N (100) /W (110) heterostructure is 0.14 eV, suggesting that H atoms are easily desorbed from the catalyst surface, resulting in the superaerophobic effect and promoting catalytic efficiency.^[^ ^24^ ^]^ In addition, the Δ*G*_H_ of the WC (100) /W_2_N (111) heterostructure was −0.16 eV, suggesting better H adsorption kinetics than WC, which is consistent with previous reports (Figures S33–S36, Supporting Information).^[^ ^22c^ ^]^ Taking into account the unchanged WC crystal phase and the high‐temperature N‐doping reaction process, and based on EPMA quantitative analysis, the actual N‐doping content should be less than 1/12 of C (Table S9, Supporting Information).^[^ ^20^, ^21^, ^41^ ^]^ Therefore, the real Δ*G*_H_ of WC‐N/W should be between −0.09 and 0.14 eV. This suggests that further optimization of their HER efficiency is needed by adjusting the content of N in order to reach a closer approach the Δ*G*_H_ = 0. The effect of vacancy and twin defects are also taken into account in the DFT calculations. The WC‐N/W heterostructure with a carbon vacancy (Figure S37, Supporting Information) and WC twins (Figure S38, Supporting Information) had a reduced Δ*G*_H_ of 0.05 eV and −0.49 eV, respectively, suggesting better HER kinetics.

The dissociation step of H_2_O in the alkaline environment is also important for HER. The free energy diagram of water dissociation process is shown in Figure 8d. In the first step, a water molecule was adsorbed on the catalyst. The next step was a transition state: the dissociation of H_2_O and finally the final state is reached. Compared to WC (100) (2.21 eV), the water dissociation energy barrier of WC (100) /W (110) (1.36 eV), WC‐N (100) /W (110) (1.04 eV), and WC (100) /W_2_N (111) (1.53 eV) were reduced. N doping favors water adsorption and dissociation to proceed at lower potential energy surfaces, which is consistent with literature reports.^[^ ^42^ ^]^ The lower reaction energy barrier suggests that the formation of the heterostructure favors the acceleration of water dissociation.

The experimental results and the theoretical calculations, in conjunction with the excellent HER catalytic performance of WC‐N/W‐1200 electrode, allow the proposal of the following overall mechanism. 1) The porous ceramic electrode with finger‐like holes exhibiting superhydrophilicity, facilitated the rapid transfer of charge and ions between the electrode and the electrolyte, and, also, accelerated the detachment of bubbles (Figure S27 and Movie S1, Supporting Information). 2) As thermal treatment temperature increased, *R*_ct_ was significantly reduced due to the increase of conductive metallic W phase; thereby, it enhanced its HER performance, which was consistent with literature related to metal/transition metal carbide (M/TMC) systems.^[^ ^18^, ^23^, ^43^ ^]^ 3) The interface of WC‐N/W heterostructure, where the electronic structure and the charge density distribution were effectively modulated, enhanced the interaction with H^+^ and the reaction kinetics of HER. 4) The stable, in situ formed, atomic conjugation between the WC‐N and W phases provided a resistance‐free path, facilitating the rapid transfer of electrons and improving reaction efficiency.^[^ ^44^ ^]^ This also leads to tight bonding between the active sites and the substrate, resulting in excellent long‐term and high‐current density stability.^[^ ^45^ ^]^ (5) The Mott‐Schottky effect in the WC‐N/W heterostructure accelerated the flow of electrons through the M/TMC interfaces until the work function reached equilibrium; thereby, it improved the electron transport efficiency and conversion rate of the catalyst in the HER reaction.^[^ ^5^, ^46^ ^]^ (6) The high temperature (in situ) reaction generated abundant defect sites that can adjust the electronic and surface properties of the electrode. As a result, it optimized the H adsorption step in electrochemical catalysis.^[^ ^47^ ^]^

## 3 Conclusions

An asymmetric porous ceramic membrane electrode for the HER process, with abundant in situ formed WC‐N/W heterostructures, was successfully prepared. As a consequence, the WC‐N/W‐1200 electrode exhibited outstanding HER performance with the lowest overpotentials of 87 and 104 mV and the smallest Tafel slopes of 44.9 and 62.2 mV dec^−1^ in acidic and alkaline media, respectively. At the same time, it maintained excellent long‐term stability at a high current density of 1000 mA cm^−2^. DFT calculation analysis attributed this extraordinary HER performance to the superhydrophilic and the structural properties of the ceramic membrane and the effect of the WC‐N/W heterostructure interface, which favor charge transport and improve catalytic efficiency. This work has a dual aim: to present a new and easy method for designing novel and efficient WC‐based HER electrocatalysts, and to produce a stable electrode which can be qualified for consideration in large‐scale industrial H_2_ production.

## 4 Experimental Section

### Synthesis of Porous WC‐N/W Heterostructure Electrodes

The porous ceramic membrane was prepared by combining phase‐inversion bilayer tape‐casting and pressureless sintering,^[^ ^17^ ^]^ as described below. First, WC and graphite slurries were prepared with the compositions shown in Table S1, Supporting Information. Briefly, polyethersulfone (PESf, Gafone 3000, Solvay Advanced Polymers) and polyvinylpyrrolidone (PVP, K30, CP, Sinopharm Chemical Reagent Co.) were dissolved in *N*‐methyl‐2‐pyrrolidone (NMP, CP, Sinopharm Chemical Reagent Co., China) to form a homogeneous polymer solution. Then, WC (0.5–1 µm, Nangong Jingrui Alloy Material Co., Ltd.) and WO_3_ (1 µm, CP, Sinopharm Chemical Reagent Co., China) or graphite (2 µm, CP, Sinopharm Chemical Reagent Co., China) powders were added to the solution, followed by ball milling at room temperature for 48 h. The obtained stable and homogeneous slurries were degassed for 20 min and then co‐tape cast on a Mylar sheet with a doctor blade with a gap height of 0.75 and 0.1 mm, for WC and C, respectively, and a speed of 40 cm min^−1^. Then, the casted slurry was immersed in water at room temperature for 12 h to coagulate. After drying, the obtained green tape was cut into rectangular shaped samples. Then, the green samples were heated at 800 °C for 3 h (the heating rate was 5 K min^−1^) to remove the polymer, and sintered at 1600 °C for 4 h in a graphite furnace under argon atmosphere. Finally, the prepared WC ceramic membrane was placed in a tubular furnace and heated in an NH_3_ atmosphere for 3 h. The sintering temperature was in the range of 1100– 1400 °C and the produced samples were denoted as WC‐N/W‐1100, WC‐N/W‐1200, WC‐N/W‐1300, and WC‐N/W‐1400. In order to compare the effect of N doping on HER performance, the WC ceramic membrane was heat‐treated at 700 °C for 3 h in CO_2_ atmosphere, and then treated at 1000 °C for 3 h in 95% Ar‐5% H_2_ atmosphere to obtain WC/W electrode.

### Materials Characterization

The crystalline phases of the samples were determined by XRD analysis (Ultima IV) using the Cu K*α* radiation. The microstructure of the samples was observed in a field emission scanning electron microscope (ZEISS, GeminiSEM 300) and a TEM (JEM‐2100F, Japan), equipped with an EDS device for elemental analysis. The chemical state of the elements at the samples’ surface was analyzed by XPS using a Model Kratos Axis supra**+** instrument with an Al K*α* X‐ray source. Raman scattering spectra were recorded by a Renishaw System 2000 spectrometer (Renishaw inVia Reflex, 514 nm). BET surface area was measured with a Tristar II model 3020 machine. EPR spectra were recorded in a JEOL JES‐FA200 EPR equipment. The qualitative analysis of the elements in the sample was carried out with an electron probe microanalyzer (EPMA‐8050G) at a voltage of 15.0 kV and a beam size of 100 µm. The samples' porosity and pore size distribution were measured using the Archimedes immersion and bubble methods.^[^ ^48^ ^]^ The mechanical strength was evaluated by three‐point flexural strength tests using an Instron equipment (Model 5567, UK; the maximum load cell was 1 kN). The flexural strength (*σ*_F_) was calculated by the formula

(6) $$\;\sigma_{F}=\frac{3FL}{2bh^{2}}$$

where *F* is the maximum force applied, and *L*, *b*, and *h* are the length, width, and thickness of the ceramic membrane, respectively.^[^ ^12^, ^48^ ^]^

The water contact angle was measured with the aid of the Attention theta (Biolin Scientific) instrument. Home‐made equipment was used to measure the permeability of water through the membrane and to evaluate the mechanical and practical characteristics of the membrane electrode in the realistic conditions of application. The ceramic membrane was encapsulated with epoxy resin on a connected mold, and then connected to a cylindrical cavity filled with water. Various pressures were applied to allow water permeation through the membrane, while an electronic balance was used to measure the weight of the permeated water.^[^ ^49^ ^]^

### Electrochemical Measurements

The electrochemical tests were conducted in a CHI760E electrochemical workstation at room temperature. A standard three‐electrode system was used for testing. The graphite rod, an electrode (Ag/AgCl and Hg/HgO of acidic and alkaline media), and the prepared ceramic membrane were the counter electrode, the reference electrode, and the working electrode, respectively. The tested media were 0.5 m H_2_SO_4_ and 1 m KOH aqueous solutions. The electrodes were immersed in the electrolyte and were conditioned by 50 cycles of potential applied between 0.0 and ≈0.5 V, with a scanning rate of 50 mV s^−1^. The electrocatalytic activity of the samples was characterized by LSV at a sweep rate of 5 mV s^−1^ under a potential window of 0–1.0 V in N_2_‐saturated testing media. All the potentials of the reversible hydrogen electrode (RHE) were calculated according to the formulae:

(7) $$E_{\mathrm{RHE}}=E_{\mathrm{Ag}/\mathrm{AgCl}}\;+0.0592\;\times \;\mathrm{PH}+0.197\;V\;\mathrm{for}\;0.5\;M\;H_{2}SO_{4}$$

and

(8) $$\;E_{\mathrm{RHE}}=E_{\mathrm{Hg}/\mathrm{HgO}}\;+0.0592\;\times \;\mathrm{PH}+0.098\;V\;\mathrm{for}\;1\;M\;\mathrm{KOH}$$

EIS was performed in a constant potential mode in the 10^5^–0.1 Hz frequency range and the voltage was fixed at −400 mV. The electrochemical active surface areas of the prepared samples were obtained from double‐layer capacitors (*C*_dl_), which were calculated using CVs with various scanning rates (5–30 mV s^−1^). The

ECSA was calculated by the following formula:

(9) $$\mathrm{ECSA}\;=C_{\mathrm{dl}}\;/C_{s}$$

where *C*_s_ is the specific capacitance with a value of 0.040 mF cm^−2^.^[^ ^39^ ^]^ The presenting curves have been calibrated with RHE after being corrected according to the IR compensation. The Faraday efficiency (FE) was calculated by the following formula:

(10) $$\mathrm{FE}\;\;\left(H_{2}\right)=\frac{2\;\times n\times F}{I\times t}\;\times \;100\%$$

where *n* represents the moles of the produced H_2_, *F* is the Faraday constant, and *I* and *t* are the current and time, respectively. Hydrogen (H_2_) was analyzed by Techcomp GC‐7900 gas chromatograph.^[^ ^12a^ ^]^ 5.0 mg Pt/C (20 wt% Pt) was dispersed in a mixed solution of 380 µL deionized water, 100 µL absolute ethanol, and 20 µL 0.5 wt% Nafion, and sonicated for 2 h to form a uniform catalyst ink. Then, 20 µL of the catalyst ink was deposited on the glassy carbon electrode with a mass loading of 1.0 mg cm^−2^.

### Theoretical DFT Calculations

The first principle calculations based on density functional theory (DFT) were performed using the Materials Studio CASTEP module. The calculations were implemented with the Perdew–Burke–Ernzerhof (PBE) exchange‐correlation functional under the generalized gradient approximation scheme with a cut off energy of 350 eV. All the structures were optimized with force exerted on the atoms less than 0.02 eV Å^−1^, and the total energy convergence was set as 1.0 × 10^−5 ^eV per atom. To optimize the heterostructure, the Brillouin‐zone was sampled by the Monkhorst–Pack grid with a density of 4 × 4 × 2. The lattice parameters of the optimized WC (*a* = *b* = 2.928 Å, *c* = 2.849 Å, *α* = *β* = 90^o^, *γ* = 120^o^) and W (*a* = *b* = *c* = 3.208 Å, *α* = *β* = *γ* = 90^o^) were basically the same with experimental values. The catalytic mechanism of the WC‐N/W heterointerface was investigated by using the 2 × 2 surface of WC (100) and W (110) to construct the WC‐N / W (N replaced 1/12 C) heterostructure based on the HRTEM images. The twin of WC crystal was constructed by two symmetrical WC (100) planes. To investigate the effect of vacancy on the performance, a WC‐N/W heterostructure with a vacancy was constructed by removing a C atom from the layer. The model of W_2_N/WC was constructed by using a 2 × 2 surface of W_2_N (111) and WC (100). In order to avoid the interaction between images, a 15 Å vacuum space was included. All the atoms and the lattice parameters of the heterostructure were fully relaxed, and the structure remained orthogonal after being optimized (Table S10, Supporting Information).

The free energy of hydrogen adsorption (Δ*G*_H_) proposed by Norskov et al. was considered the key parameter for determining HER activity.^[^ ^40^ ^]^ The models of H adsorption on WC (100) /W (110) (N replaced 0 C), WC‐N (100) /W (110) (N replaced 1/12 C) and WC (100), WC (100) /W_2_N (111), WC‐N (100) /W (110) (N replaced 1/12 C, vacancy) and WC crystal twin were optimized to calculate their adsorption free‐energy (Figure 8; Figures S32, S36–S38, and Table S10, Supporting Information). The adsorption free‐energy was calculated by the equations

(11) $$\Delta E_{H}=E_{\mathrm{ads}}\;-1/2E_{H_{2}}-E_{\mathrm{WC}-N/W}$$

(12) $$\Delta G_{H}=\Delta E_{H}+\Delta \mathrm{ZPE}-T\Delta S_{H}$$

where Δ*E*_H_ is the H adsorption energy, *E*_ads_ represents the total energy of H adsorption on the surface of catalysts, *E*_H2_ and *E*_WC‐N/W_ are the energy of Hydrogen molecule and heterostructure, respectively. Δ*G*_H_ is the free energy of H adsorption, ΔZPE and Δ*S*_H_ are zero‐point energy change and entropy change of H adsorption. The value of ΔZPE−*T*Δ*S*_H_ is 0.24 eV.^[^ ^40^ ^]^ The water dissociation process and dissociation energy barrier were computed by searching transition states using PBE.

## Conflict of Interest

The authors declare no conflict of interest.

## Supporting information

Supporting Information

Supplemental Movie 1

## Data Availability

The data that support the findings of this study are available from the corresponding author upon reasonable request.
