# Supplementary material for: Robust Porous WC‐Based Self‐Supported Ceramic Electrodes for High Current Density Hydrogen Evolution Reaction
Source: Adv Sci (Weinh). 2022 Mar 25;9(15):2106029. doi: 10.1002/advs.202106029 (PMC9130889; doi:10.1002/advs.202106029)
Supplement: Supplementary file 1 — Supporting Information [file ADVS-9-2106029-s001.pdf]

ADVANCED SCIENCE
Open Access

Supporting Information

for *Adv. Sci.*, DOI 10.1002/advs.202106029

Robust Porous WC-Based Self-Supported Ceramic Electrodes for High Current Density Hydrogen Evolution Reaction

Feihong Wang, Yutong Wu, Binbin Dong, Kai Lv, Yangyang Shi, Nianwang Ke, Luyuan Hao, Liangjun Yin, Yu Bai, Xin Xu, Yuxi Xian* and Simeon Agathopoulos*

Supporting Information

Robust Porous WC-based Self-Supported Ceramic Electrodes for High Current Density Hydrogen Evolution Reaction

Feihong Wang[1,+], Yutong Wu[1,+], Binbin Dong[2], Kai Lv[1], Yangyang Shi[1], Nianwang Ke[1], Luyuan Hao[1], Liangjun Yin[3], Yu Bai[4], Xin Xu[1,], Yuxi Xian[5,*], and Simeon Agathopoulos[6]*

[1] CAS Key Laboratory of Materials for Energy Conversion, Department of Materials Science and Engineering, University of Science and Technology of China, Hefei, Anhui 230026, PR China.

[2] School of Materials Science and Engineering, Henan Key Laboratory of Special Protective Materials, Luoyang Institute of Science and Technology, Luoyang, Henan 471023, PR China

[3] School of Energy Science and Engineering, University of Electronic Science and Technology of China, 2006 Xiyuan Road, Chengdu, PR China.

[4] School of Engineering Science, University of Science and Technology of China, Hefei, Anhui 230026, PR China.

[5] CAS Key Laboratory of Mechanical Behaviors and Design of Materials, Department of Modern Mechanics, University of Science and Technology of China, Hefei, Anhui 230026, PR China

[6] Department of Materials Science and Engineering, University of Ioannina, GR-451 10 Ioannina, Greece.

[*]: Corresponding author: Tel.: +86 551 3600824, Fax: +86 551 3601592

E-mail address: xuxin@ustc.edu.cn (Prof. Xin Xu)

[+] These authors contributed equally to this work.

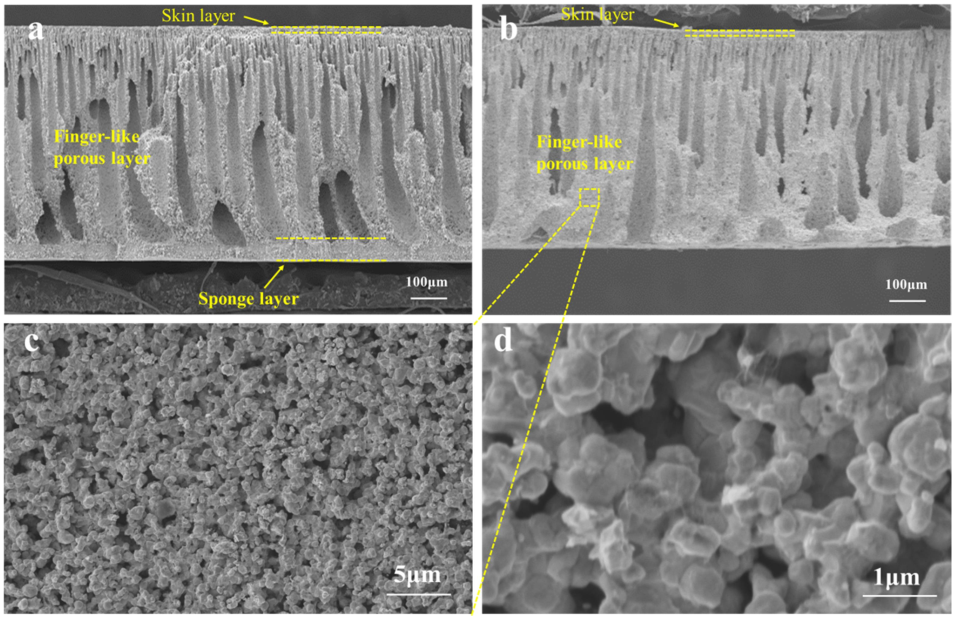

Figure S1. Microstructure of the cross-section of ceramic membranes, a) the green sample, and b-d) after heat treatment at 1600 ^{o}C in Ar atmosphere at various magnifications.

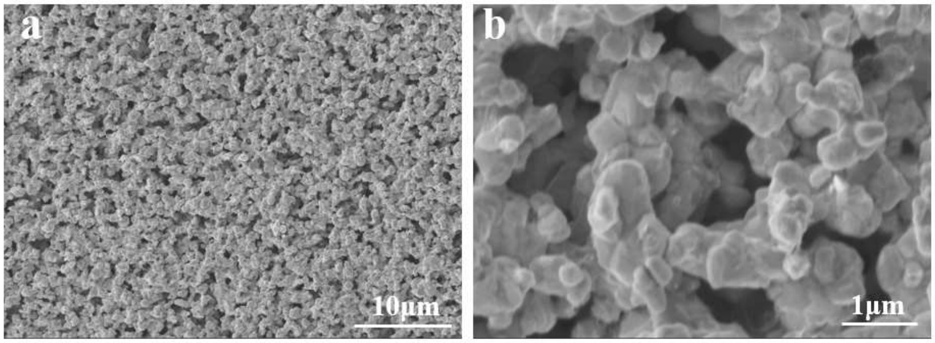

Figure S2. Microstructure of the skin layer surface of WC-N/W-1200 electrode at a) low and b) high magnification.

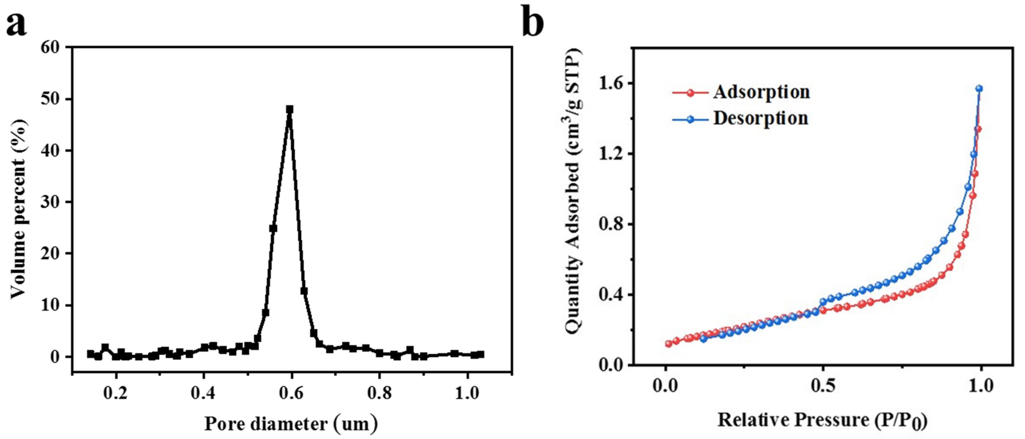

Figure S3. a) Pore size distribution and b) BET isotherms of WC-N/W-1200 electrode.

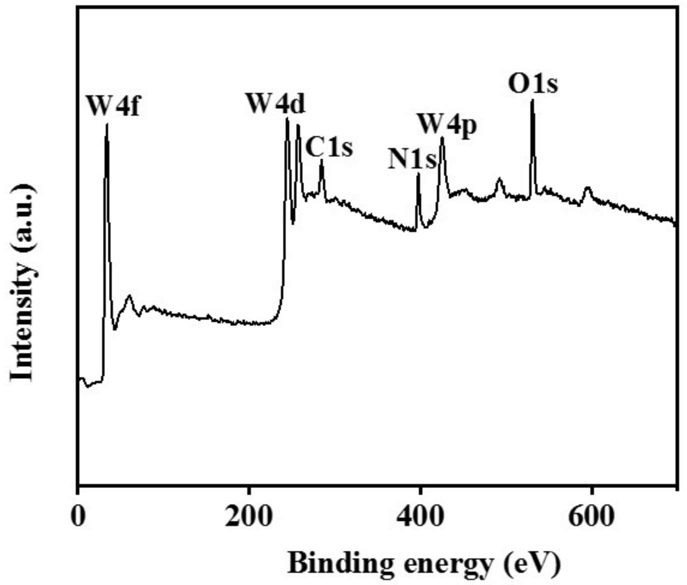

Figure S4. XPS full-spectrum of WC-N/W-1200 electrode.

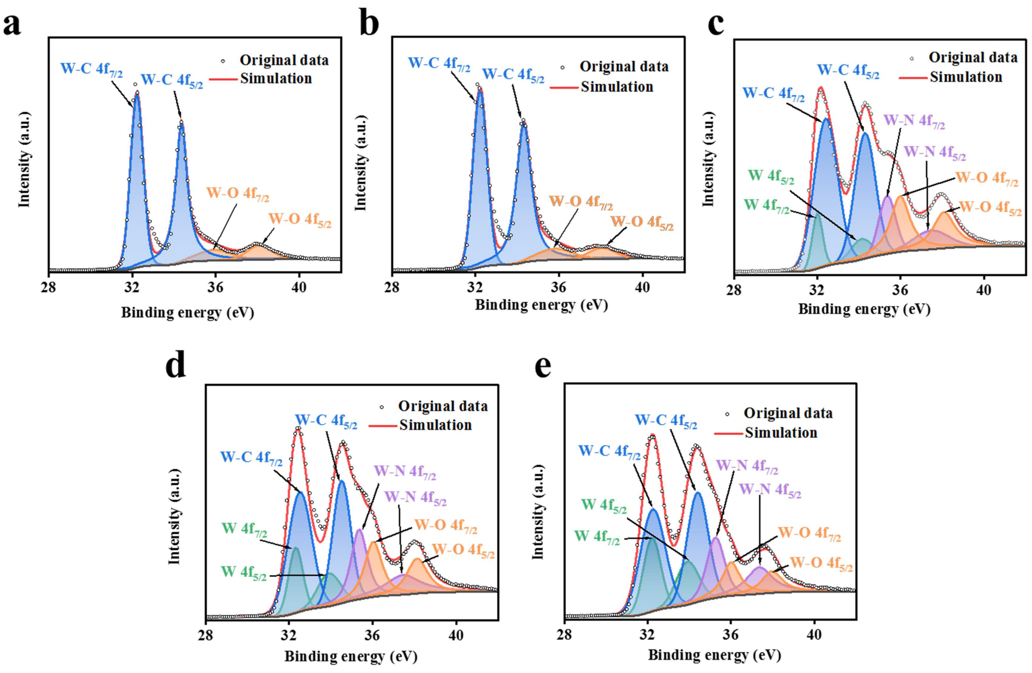

Figure S5. High resolution XPS spectra of W 4f of a) WC, b) WC-N/W-1100, c) WC-N/W-1200, d) WC-N/W-1300, and e) WC-N/W-1400.

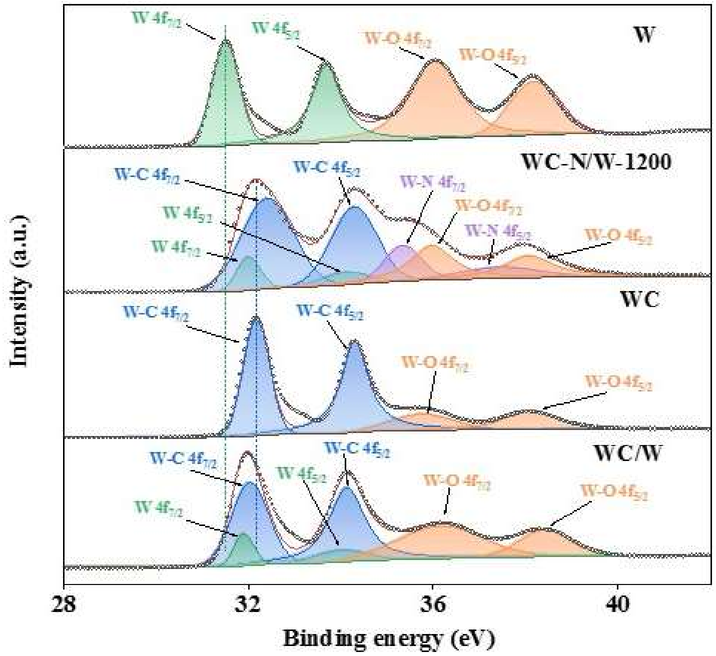

Figure S6. High resolution XPS spectra of W 4f in W, WC, WC-N/W-1200 and WC/W.

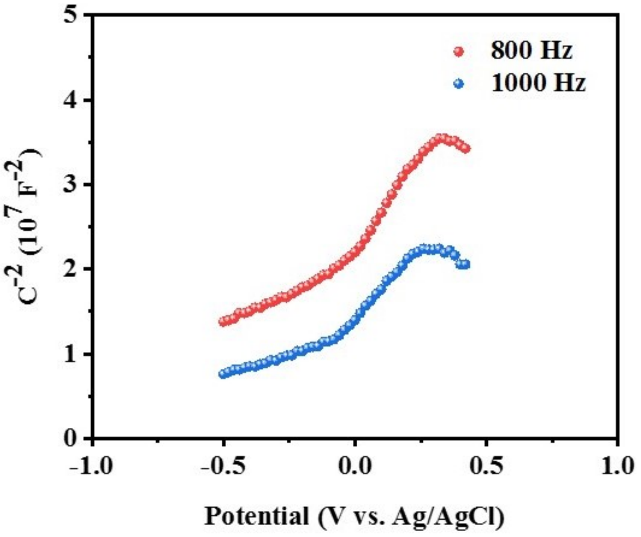

Figure S7. Mott-Schottky plots of WC-N/W-1200 electrode.

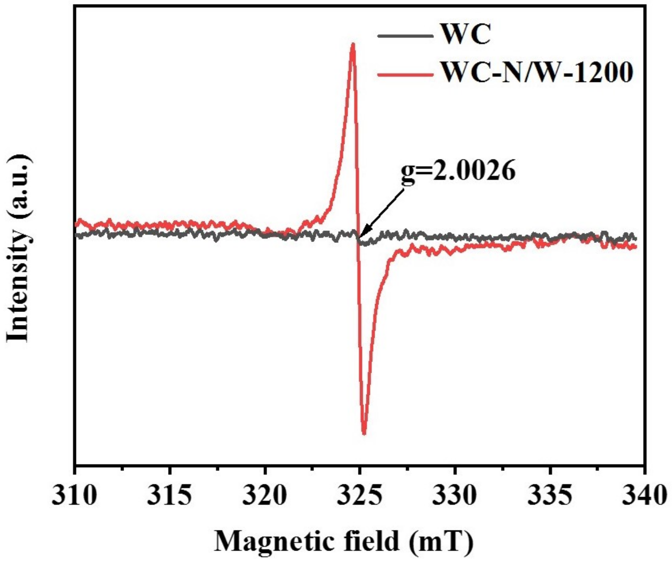

Figure S8. Electron paramagnetic resonance (EPR) spectra of WC and WC-N/W-1200 electrodes.

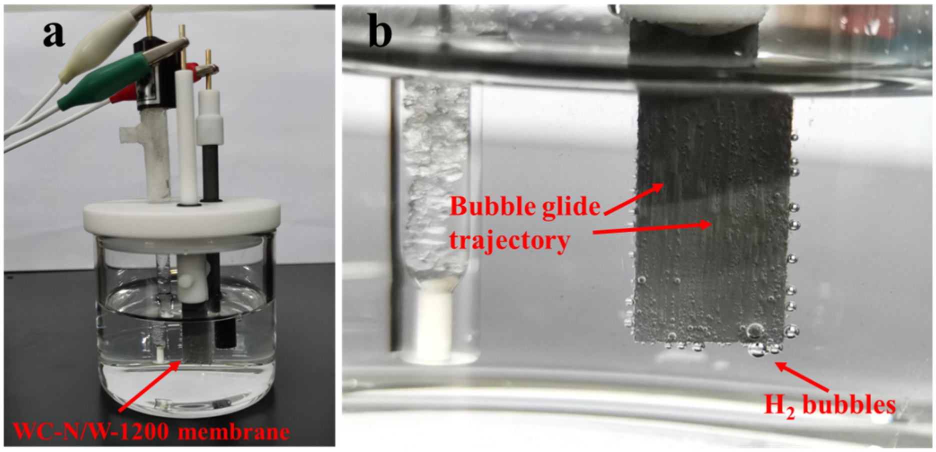

Figure S9. (a) Electrochemical test device and (b) H_2 evolved from the electrode in acidic media.

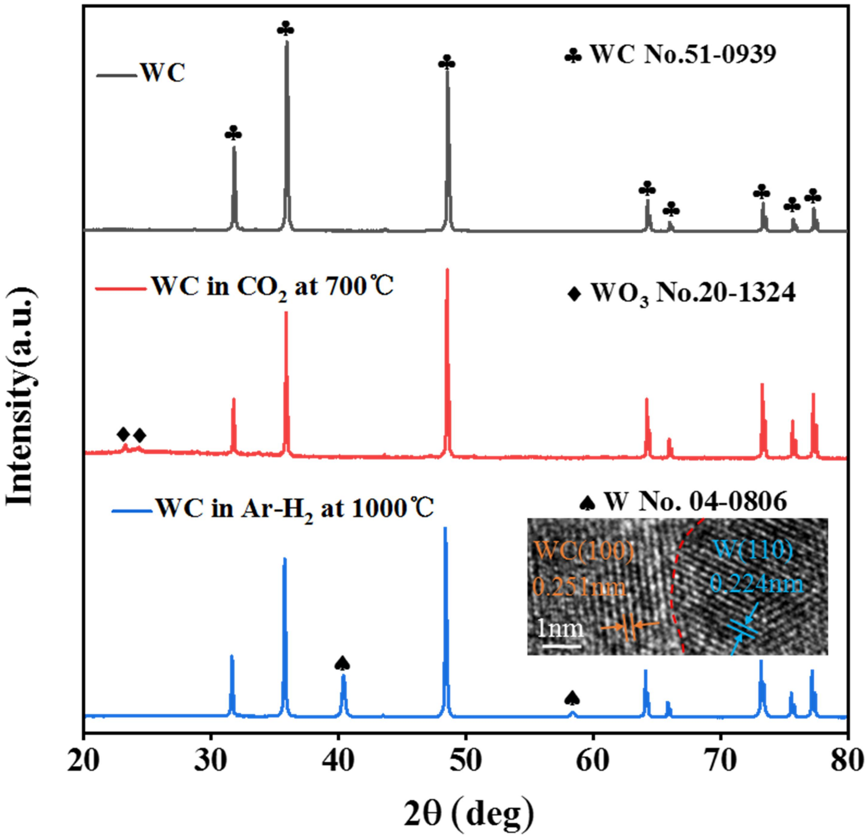

Figure S10. X-ray diffractograms of WC and WC electrodes after heat treatment in CO_2 and Ar-H_2 atmosphere (the inset illustrates the HRTEM image).

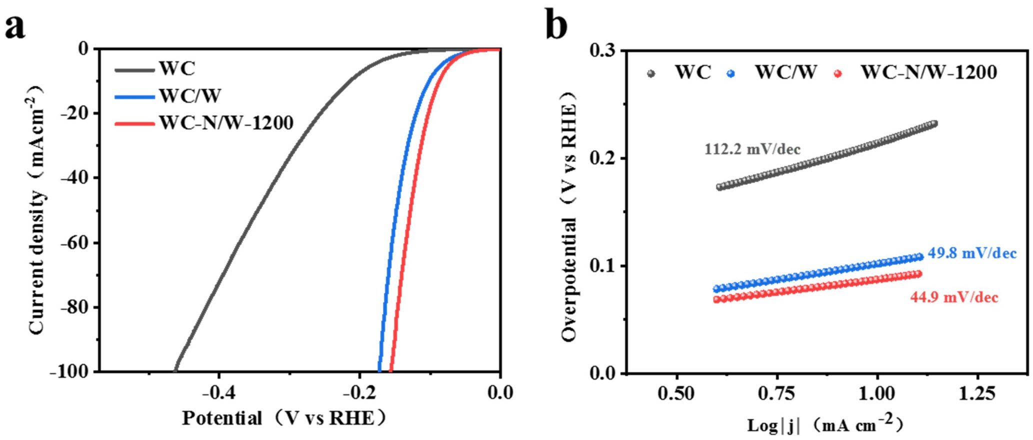

Figure S11. Comparison of a) LSV and b) Tafel curves of WC, WC/W and WC-N/W-1200 electrodes in 0.5 M H_2SO_4.

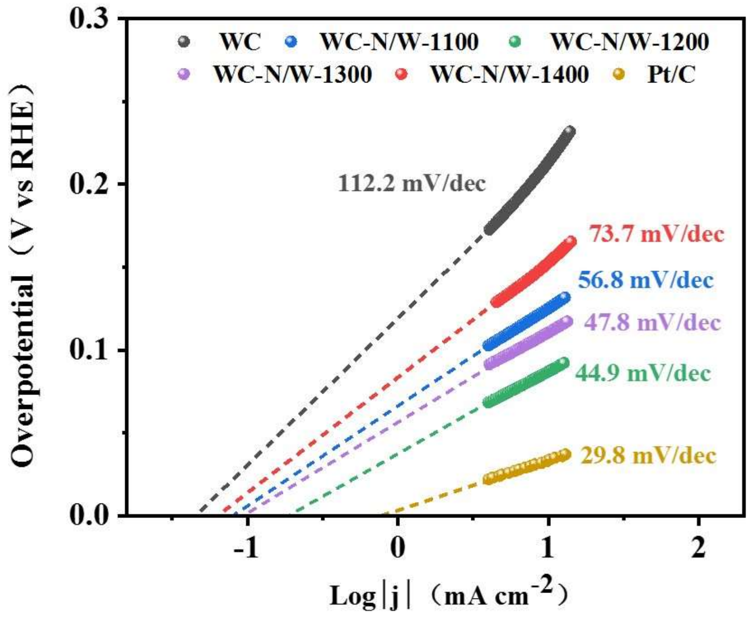

Figure S12. Exchange current density (j_0) of the WC and the WC-N/W-*T* electrodes calculated by applying extrapolation method for HER in 0.5 M H_2SO_4.

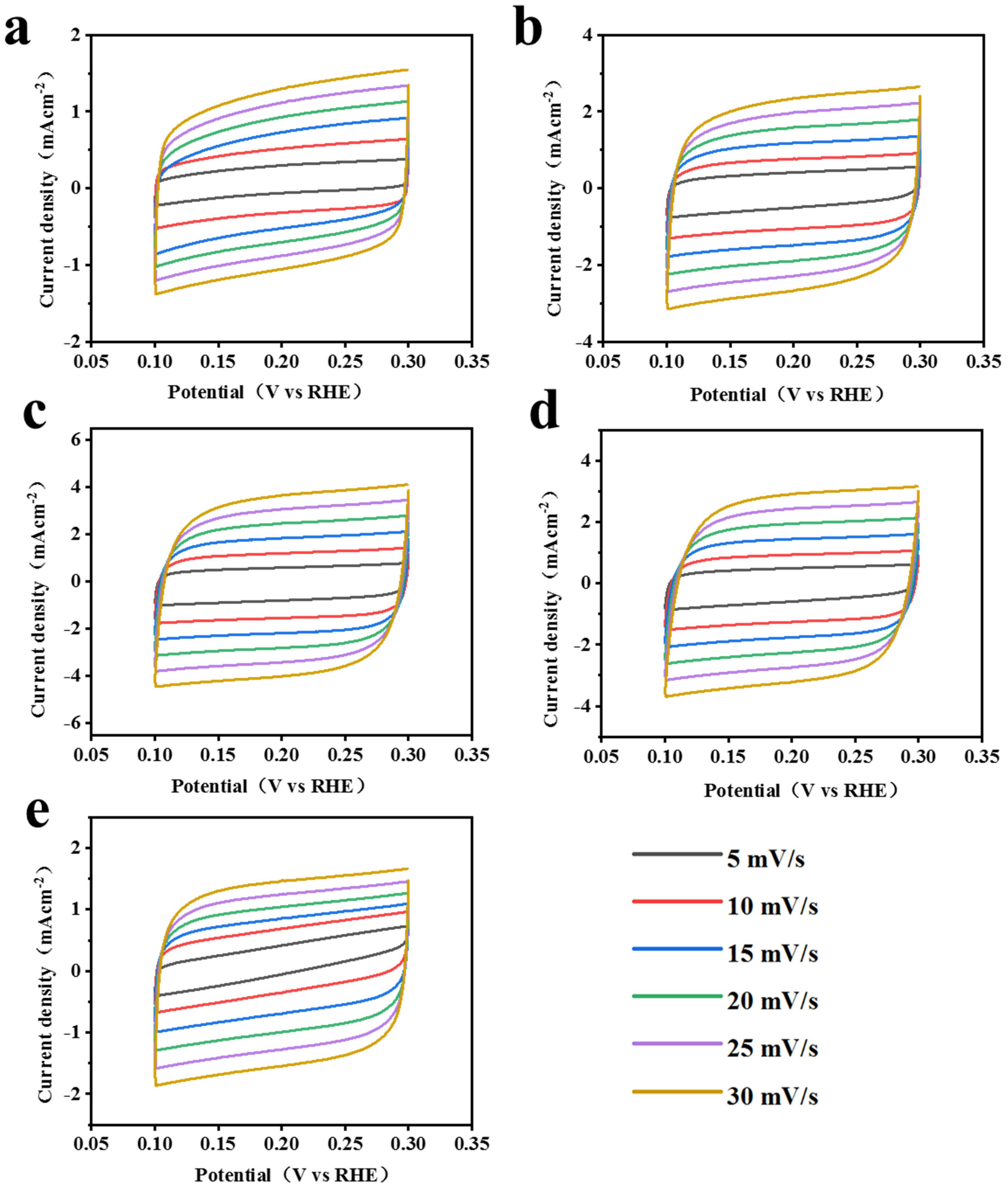

Figure S13. The CV curves of a) WC, b) WC-N/W-1100, c) WC-N/W-1200, d) WC-N/W-1300, and e) WC-N/W-1400 electrodes in 0.5 M H_2SO_4 for evaluating the C_{dl}.

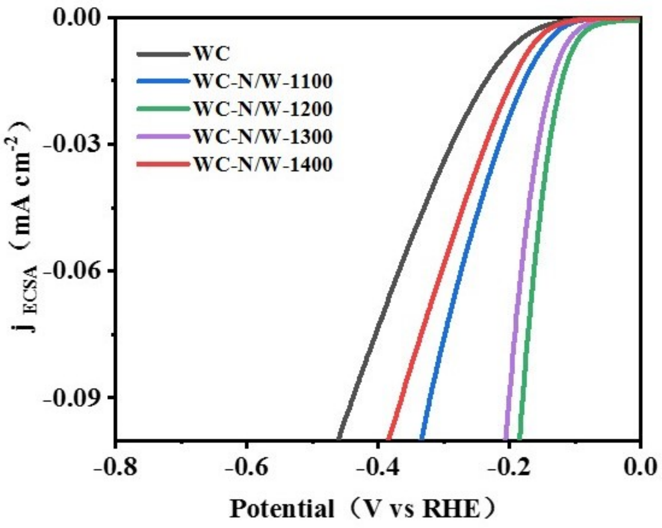

Figure S14. ECSA normalized LSV curves of the prepared electrodes in 0.5 M H_2SO_4.

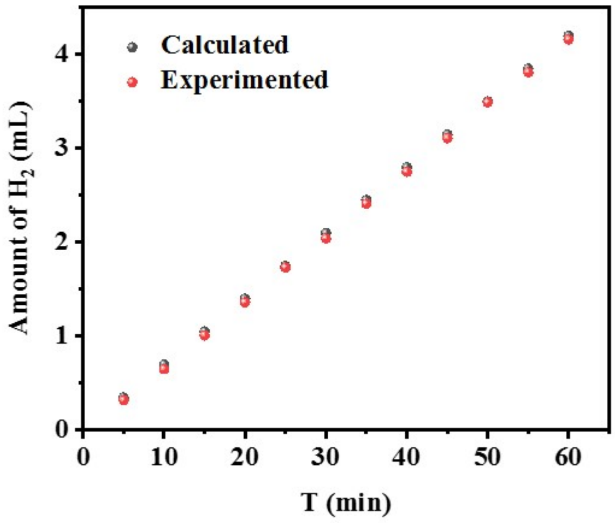

Figure S15. Faraday efficiency of WC-N/W-1200 in 0.5 M H_2SO_4.

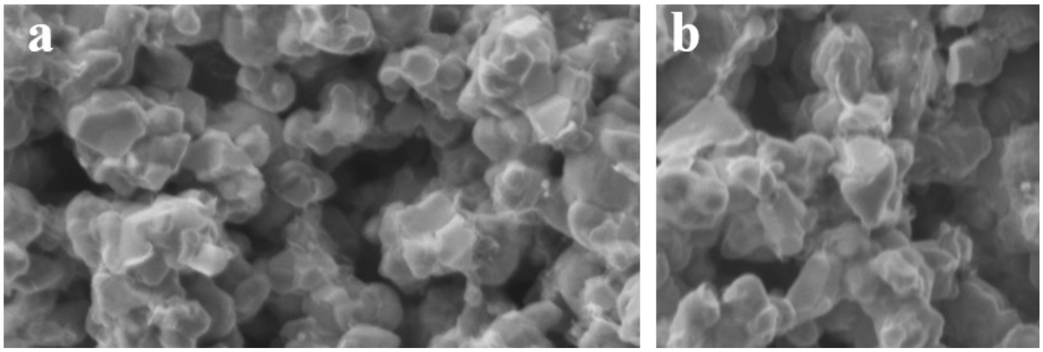

Figure S16. Microstructure of the WC-N/W-1200 electrode after cycling in a) 0.5 M H_2SO_4

and b) 1 M KOH.

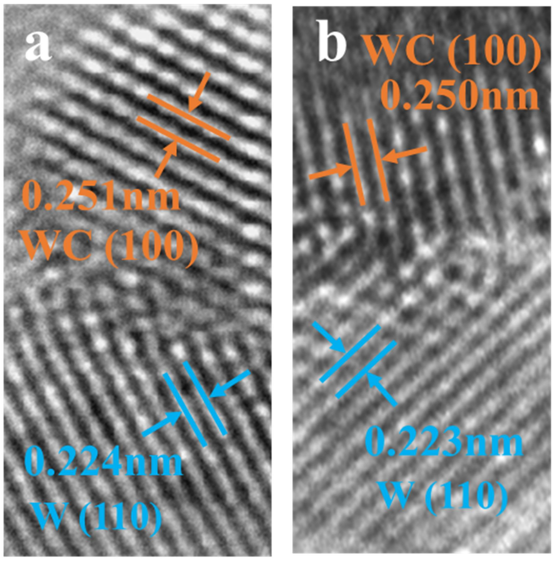

Figure S17. HRTEM images of the WC-N/W-1200 electrode after cycling in a) 0.5 M H_2SO_4 and b) 1 M KOH.

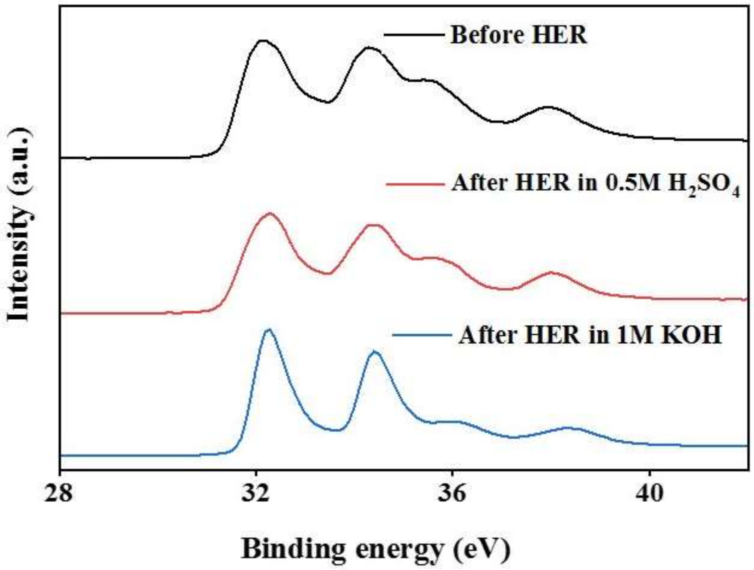

Figure S18. XPS spectra of the WC-N/W-1200 membrane after cycling in 0.5 M H_2SO_4 and 1 M KOH.

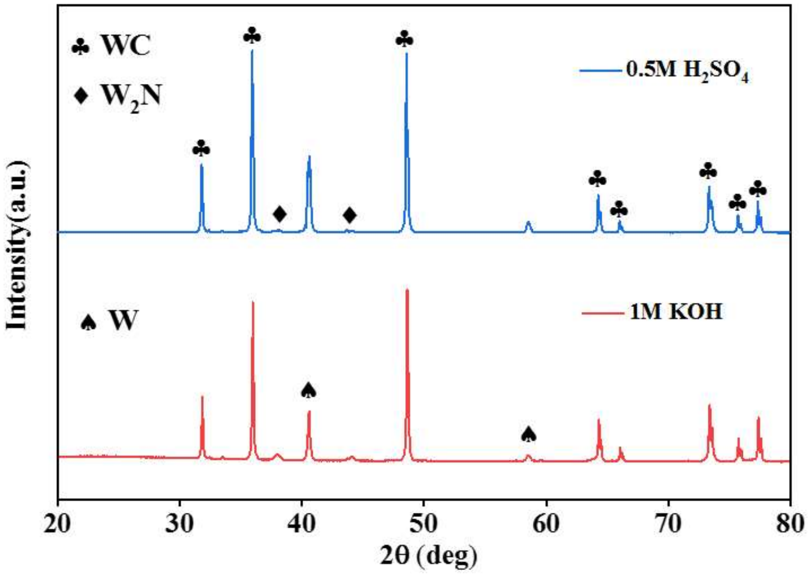

Figure S19. X-ray diffractograms of the WC-N/W-1200 membrane after cycling in 0.5 M H_2SO_4 and 1 M KOH.

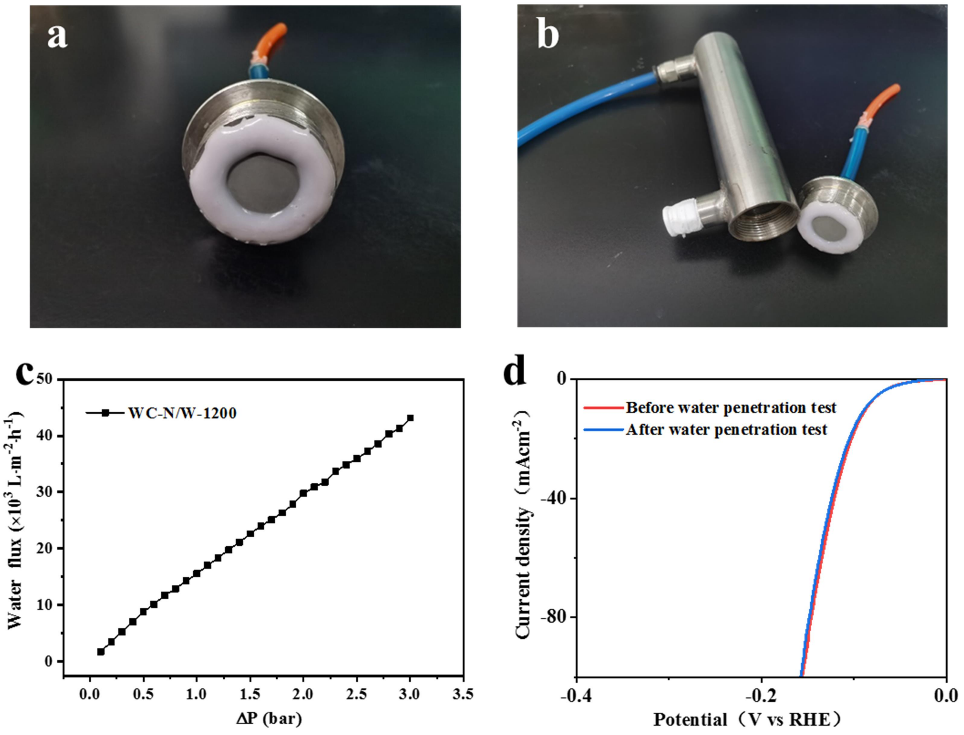

Figure S20. Water permeability of a) the encapsulation membrane, b) the test device, c) water permeability curve, and d) LSV curves of the WC-N/W-1200 electrode before and after 4 h penetration test in 0.5 M H_2SO_4.

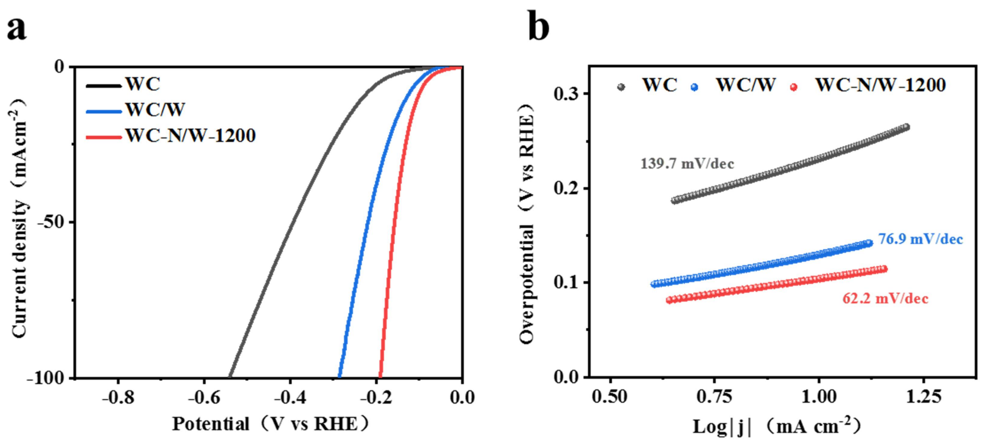

Figure S21. Comparison of a) LSV and b) Tafel curves of WC, WC/W, and WC-N/W-1200 electrodes in 1 M KOH.

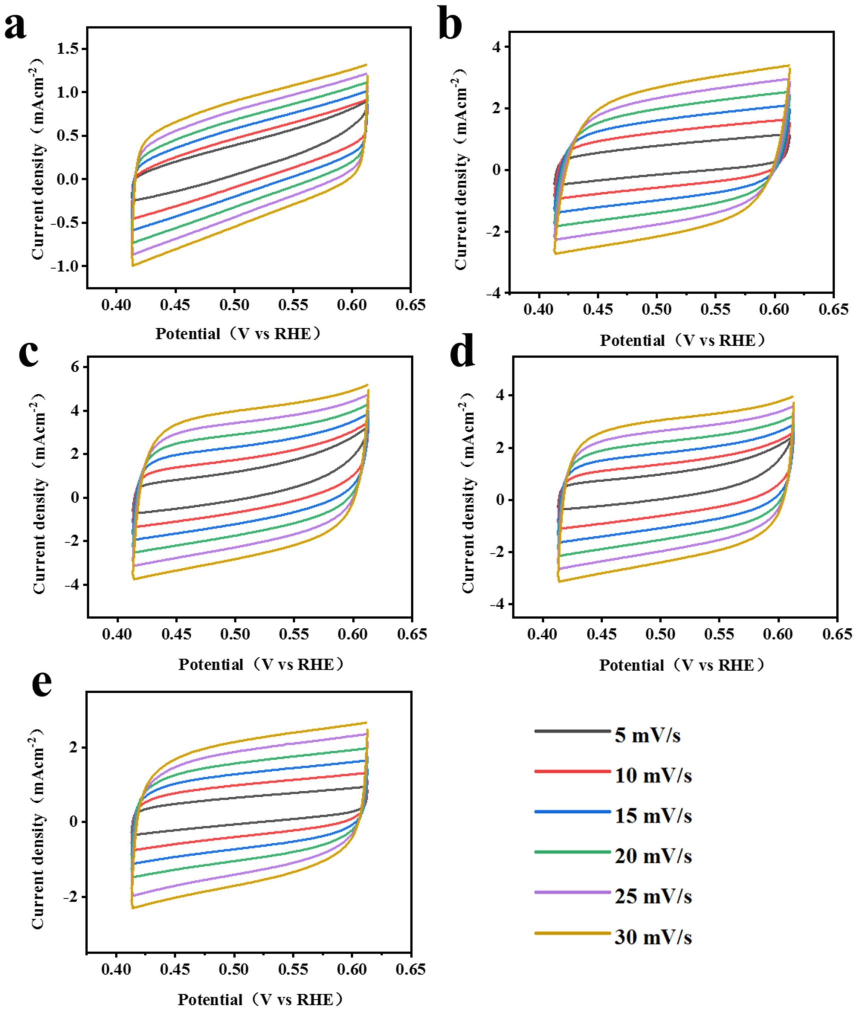

Figure S22. The CV curves of a) WC, b) WC-N/W-1100, c) WC-N/W-1200, d) WC-N/W-1300, and e) WC-N/W-1400 electrodes in 1 M KOH for evaluating the C_{dl}.

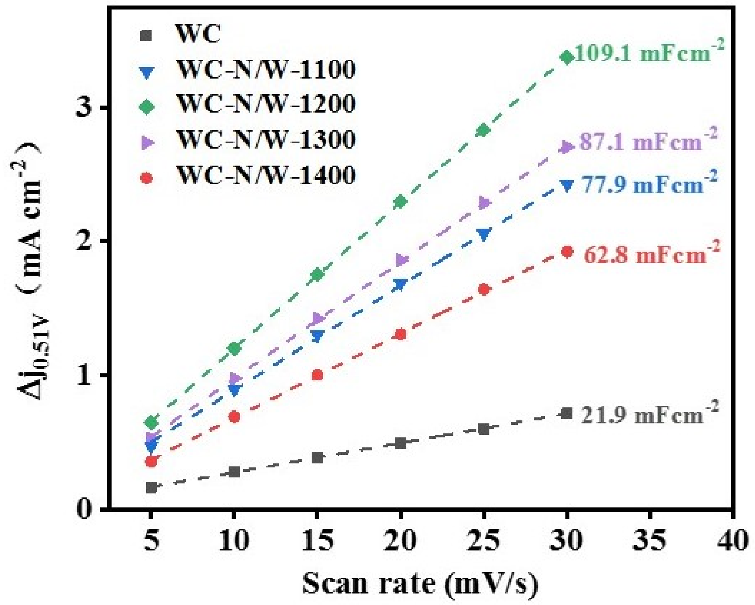

Figure S23. C_{dl} values at the potential of 0.51 V (vs. RHE) of WC and WC-N/W-T membrane electrodes in 1 M KOH.

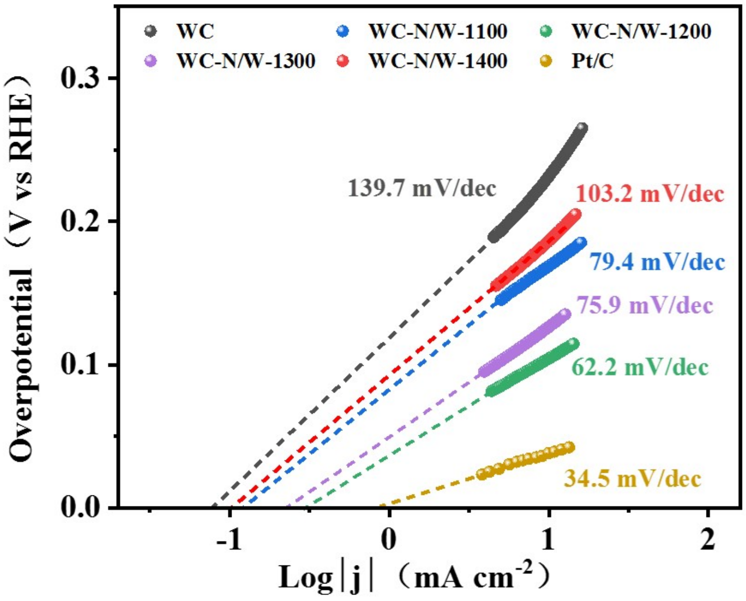

Figure S24. Exchange current density (j_0) of WC and WC-N/W electrodes calculated by applying extrapolation method for HER in 1 M KOH.

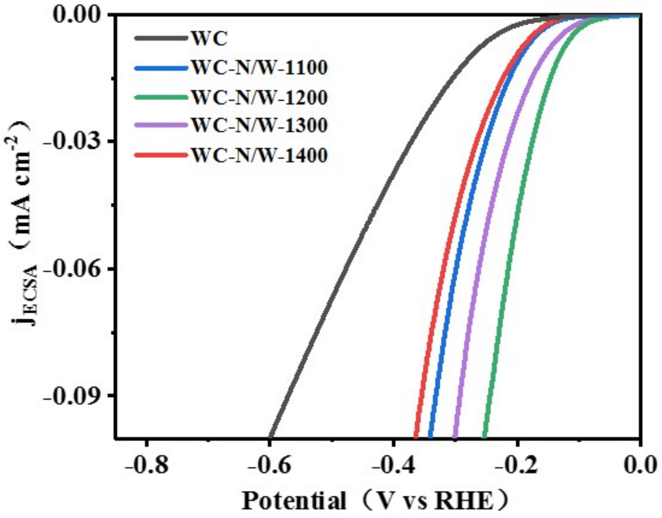

Figure S25. ECSA normalized LSV curves of prepared electrodes in 1 M KOH.

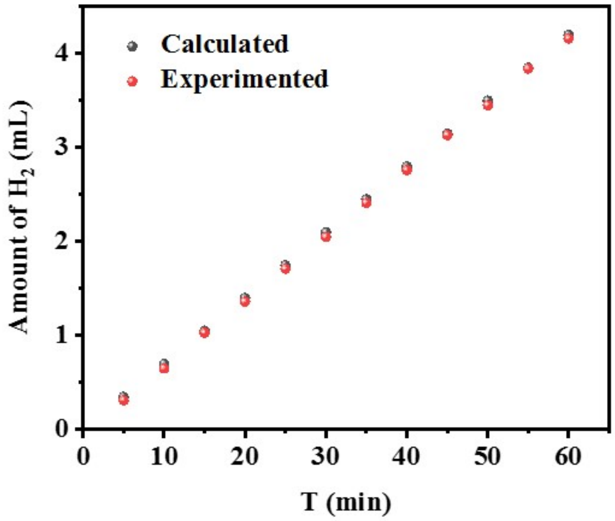

Figure S26. Faraday efficiency of WC-N/W-1200 in 1 M KOH.

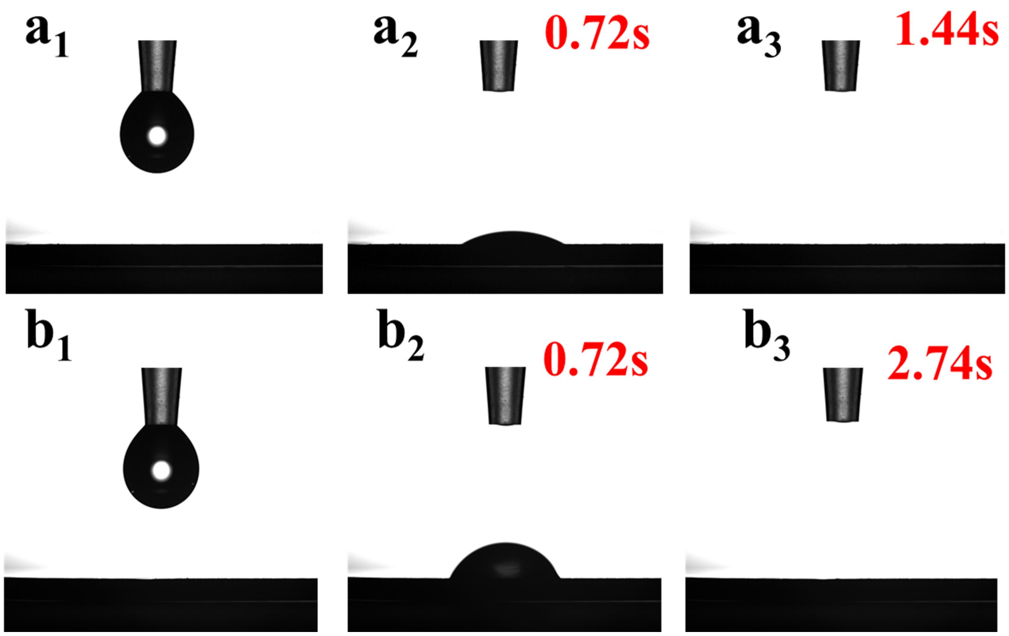

Figure S27. Water contact angle evolution over time for the WC-N/W-1200 membrane in a) H_2SO_4 and b) KOH.

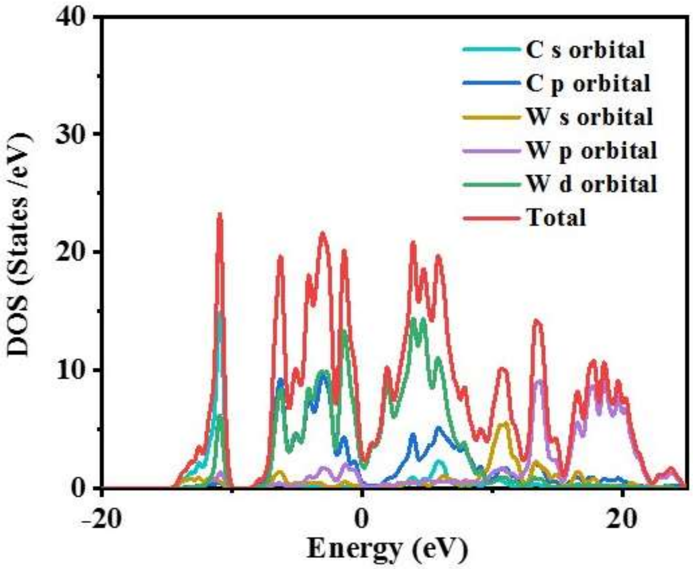

Figure S28. The density of states (DOS) for W and C atoms in WC (100).

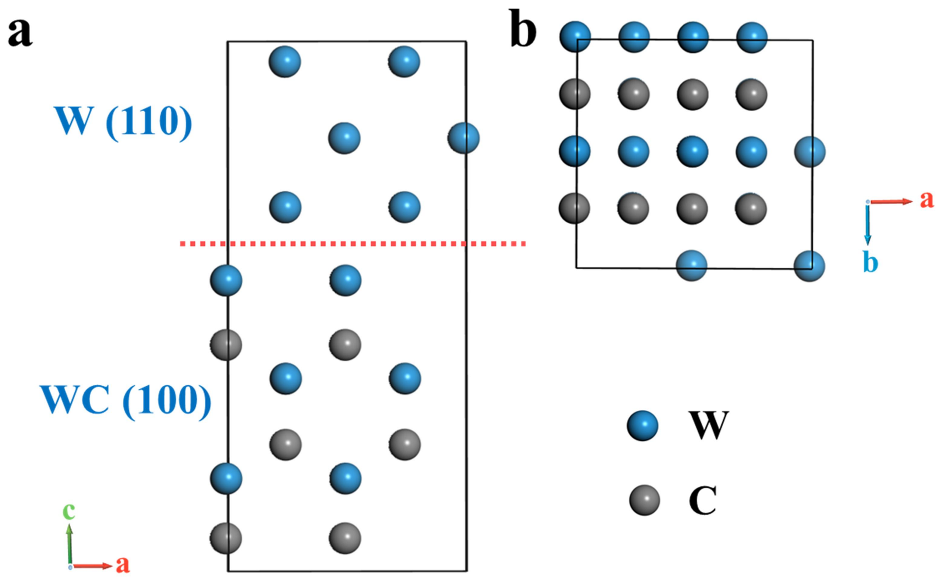

Figure S29. a) Side and b) bottom view of the WC (100) /W (110) heterostructure model.

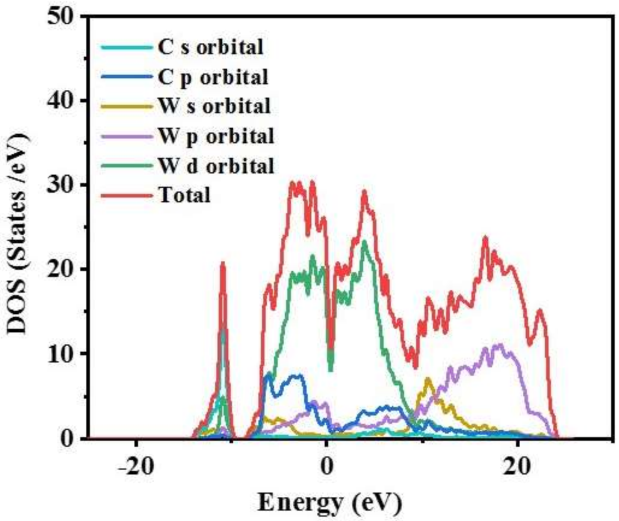

Figure S30. The density of states (DOS) for W and C atoms in WC (100) /W (110) heterostructure.

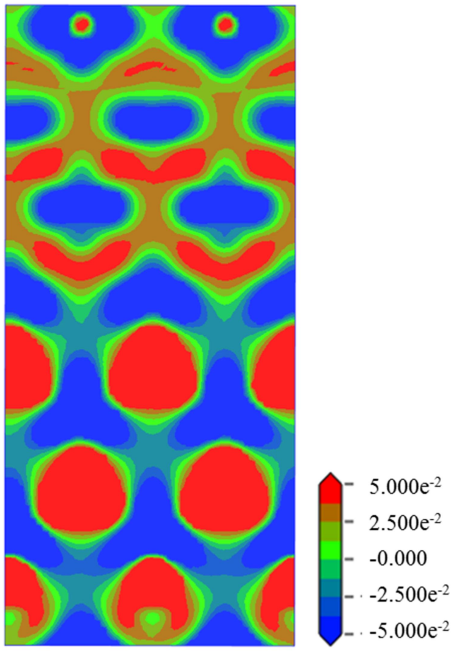

Figure S31. Slice of charge density difference at the W/WC interface in WC (100)/W(110) heterostructure.

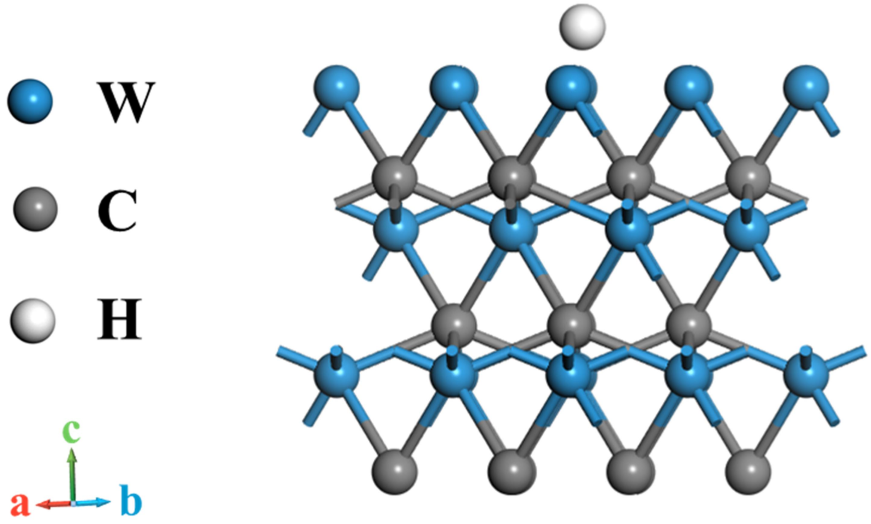

Figure S32. Optimized structure of adsorbed H on the WC (100) model surface.

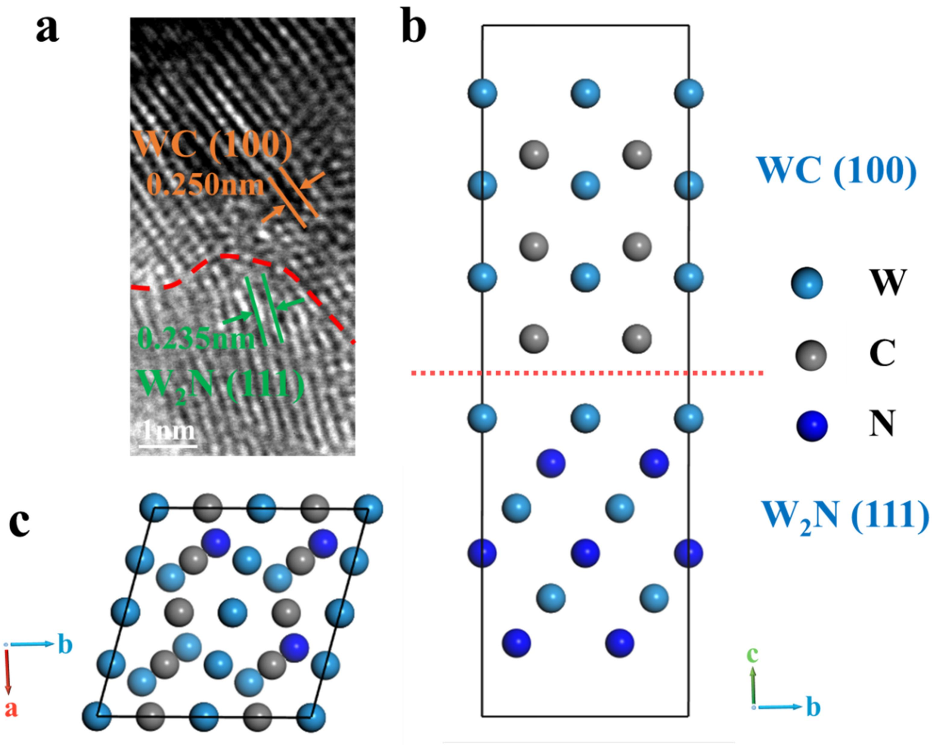

Figure S33. a) HRTEM image of WC (100) /W_2N (111) heterostructure, and b) side and c) bottom view of the WC (100) /W_2N (111) heterostructure model.

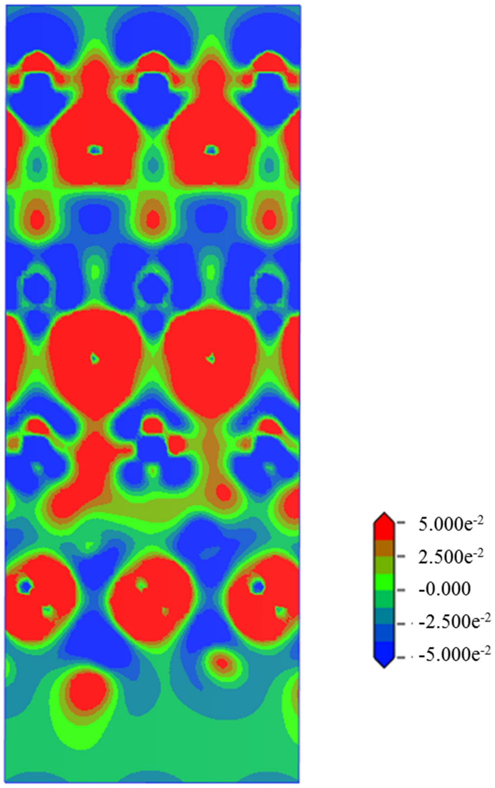

Figure S34. Slice of charge density difference at the W_2N/WC interface in WC (100) /W_2N (111) heterostructure.

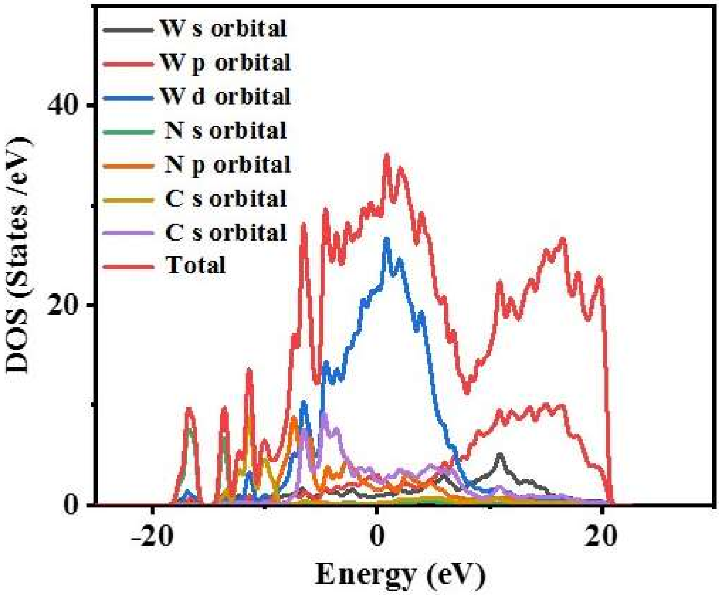

Figure S35. The density of states (DOS) for W, N, and C atoms in WC (100) /W_2N (111) heterostructure.

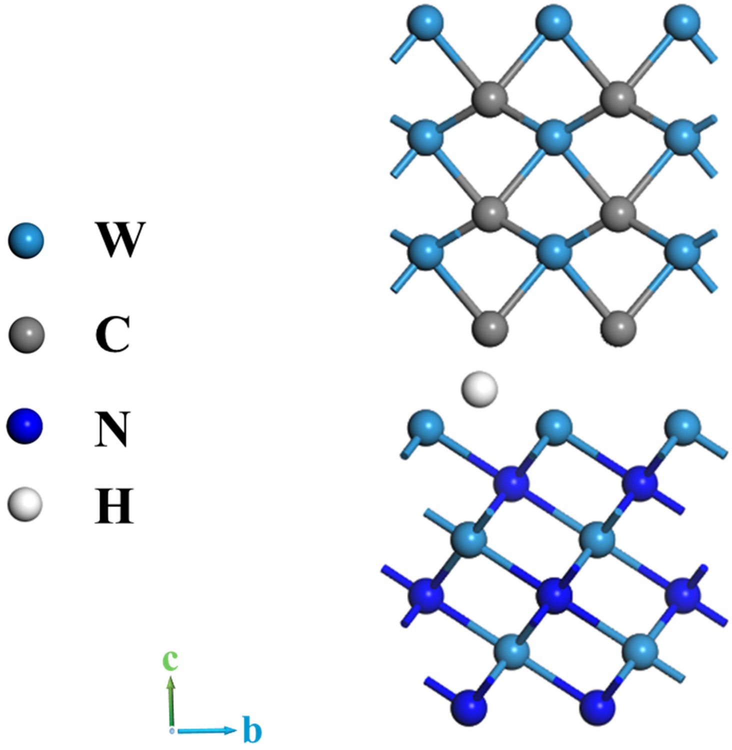

Figure S36. Optimized structure of adsorbed H on the WC (100) /W_2N (111) heterostructure model.

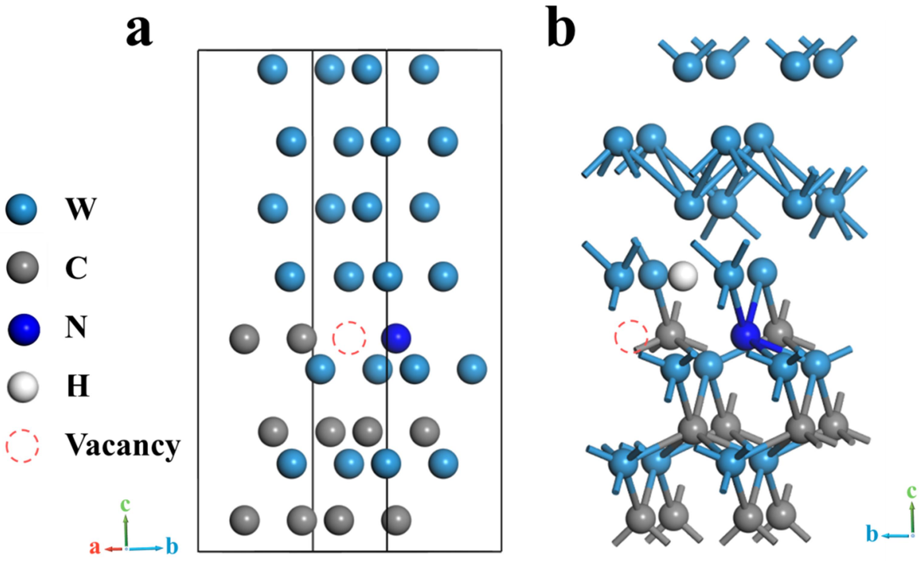

Figure S37. a) Schematic representation of the model of WC-N (100) /W (110) (N replaced 1/12 C) heterostructure with vacancy, and b) optimized structure of adsorbed H on the heterostructure models.

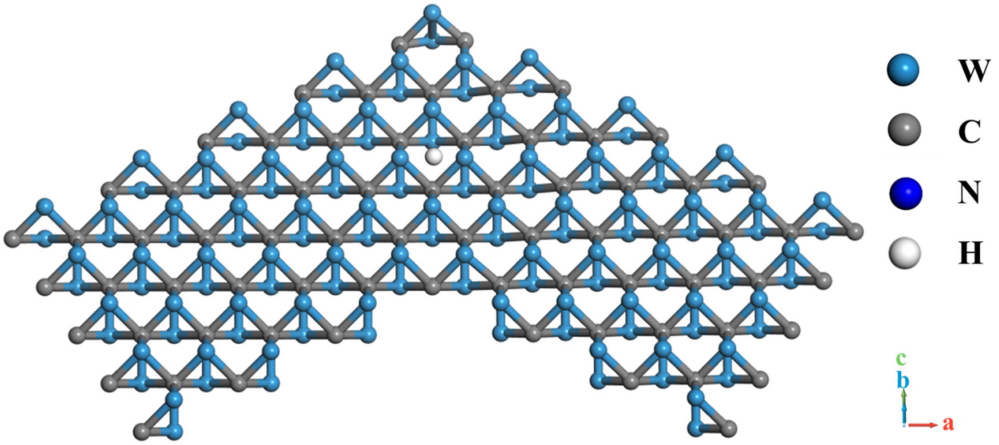

Figure S38. Structure of WC(100) twin adsorption of H.

Table S1. Composition of WC and graphite slurries (in wt.%).

Composition	WC Slurry	Graphite Slurry
**N-methyl-2-pyrrolidone (NMP)**	16.8	55.0
**Polyethersulfone (PESf)**	4.9	11.0
**Polyvinylpyrrolidone (PVP)**	1.0	1.7
**WC powder**	73.6	None
**WO_3 powder**	3.7	None
**Graphite powder**	None	32.3

Table S2. HER performance of the prepared WC and WC-N/W-T membrane electrodes in 0.5 M H_2SO_4 media.

Samples	Overpotential (mV)	Tafel slope (mV/dec)	Cdl (mF cm^{-2})	j_0 (mA cm^{-2})	R_{ct} (Ω)
**WC**	213	112.2	39.4	0.046	4.618
**WC-N/W-1100**	126	56.8	81.7	0.083	0.817
**WC-N/W-1200**	87	44.9	125.3	0.192	0.557
**WC-N/W-1300**	111	47.8	100.3	0.096	0.586
**WC-N/W-1400**	152	73.7	49.7	0.066	2.330

Table S3. ECSA of the prepared WC and WC-N/W-T membrane electrodes in 0.5 M H_2SO_4 and 1 M KOH.

Samples	A_{ECSA} (cm^2) 0.5 M H_2SO_4	A_{ECSA} (cm^2) 1 M KOH
**WC**	985.0	547.5
**WC-N/W-1100**	2042.5	1947.5
**WC-N/W-1200**	3132.5	2727.5
**WC-N/W-1300**	2507.5	2177.5
**WC-N/W-1400**	1242.2	1570.0

Table S4. HER performance of the prepared WC and WC-N/W-T membrane electrodes in 1 M KOH media.

Samples	Over potential (mV)	Tafel slope (mV/dec)	C_{dl} (mF cm^{-2})	j_0 (mA cm^{-2})	R_{ct} (Ω)
**WC**	232	139.7	21.9	0.080	6.923
**WC-N/W-1100**	168	79.4	77.9	0.122	1.123
**WC-N/W-1200**	104	62.2	109.1	0.311	0.514
**WC-N/W-1300**	126	75.9	87.1	0.229	0.861
**WC-N/W-1400**	186	103.2	62.8	0.103	2.361

Table S5. Comparison of the HER performance of the electrodes prepared in the present study with previously reported values for various tungsten carbide-based catalysts in 0.5 M H_2SO_4.

Materials	Electrolyte	Overpotential (mV)	Tafel slope (mV dec^{-1})	Ref.
**WC-CNTs**	0.5 M H_2SO_4	145	72	[1]
**2D WC**	0.5 M H_2SO_4	120	38	[2]
**N-doped WC**	0.5 M H_2SO_4	113	75	[3]
**ES-WC/W_2C**	0.5 M H_2SO_4	159	45	[4]
**WC/W_2C**	0.5 M H_2SO_4	69	52	[5]
**Cu@WC**	0.5 M H_2SO_4	92	50.5	[6]
**P-W_2C@NC**	0.5 M H_2SO_4	89	53	[7]
**W_2C/MWNT**	0.5 M H_2SO_4	123	45	[8]
**WCN**	0.5 M H_2SO_4	128	65	[9]
**WC-N/W-1200**	0.5 M H_2SO_4	87	44.9	This work

Table S6. Comparison of the HER performance of the electrodes prepared in the present study with previously reported values for various tungsten carbide-based catalysts in 1 M KOH.

Materials	Electrolyte	Overpotential (mV)	Tafel slope (mV dec^{-1})	Ref.
**WC-CNTs**	1 M KOH	137	106	[1]
**Cu@WC**	1 M KOH	119	88.7	[6]
**Co_2P/WC@NC**	1 M KOH	180	90	[10]
**W_2N/WC**	1 M KOH	148.5	47.4	[11]
**p-WC_x NWs**	1 M KOH	122	56	[12]
**$(Mo_2C)_{0.34}$-$(WC)_{0.32}$/NG**	1 M KOH	93	54	[13]
**W-W_2C**	1 M KOH	147	51	[14]
**C-CWC**	1 M KOH	73	25	[15]
**Mo_2C/W_2C**	1 M KOH	132	76	[16]
**WC-N/W-1200**	1 M KOH	104	62.2	This work

Table S7. Comparison of the HER performance of the electrodes prepared in the present study with previously reported values for metal carbide catalysts in 0.5 M H_2SO_4.

Materials	Electrolyte	Overpotential (mV)	Tafel slope (mV dec^{-1})	Syn-thesis	electrode type	Ref.
**MoC-Mo_2C-790**	0.5 M H_2SO_4	114	62	Electrolytic deposition (in situ)	Self-supported	[17]
**Mo_2N–Mo_2C/HGr**	0.5 M H_2SO_4	157	55	Catalytic etching (ex-situ)	Powder	[18]
**Mo_xC-0.4**	0.5 M H_2SO_4	155	48	Template method (ex-situ)	Powder	[19]
**W-SiC**	0.5 M	286	/	PDC	Self-	[20]

	H_2SO_4			(in situ)	supported	
**SiMoCP**	0.5 M H_2SO_4	88	37	Hydrothermal, annealing (ex-situ)	Powder	[21]
**Ti_2CT_x**	0.5 M H_2SO_4	170	100	Liquid etching (ex-situ)	Powder	[22]
**Ti_3C_2**	0.5 M H_2SO_4	169	97	Liquid etching (ex-situ)	Powder	[23]
**MoCN-3D**	0.5 M H_2SO_4	87	51.4	Hydrothermal annealing (ex-situ)	Powder	[24]
**Mo_{10}/Ti**	0.5 M H_2SO_4	180	91	Liquid etching, Hydrothermal (ex-situ)	Powder	[25]
**WC-N/W-1200**	0.5 M H_2SO_4	87	44.9	Atmosphere sintering (in-situ)	Self-supported	This work

Table S8. Comparison of the HER performance of the electrodes prepared in the present study with previously reported values for metal carbide catalysts in 1 M KOH.

**Materials**	**Electrolyte**	**Overpotential (mV)**	**Tafel slope (mV dec^{-1})**	**Synthesis**	**electrode type**	**Ref.**
**MoC–Mo_2C-790**	1 M KOH	98.2	59	Electrolytic deposition (in situ)	Self-supported	[17]
**Mo_2N–Mo_2C/HGr**	1 M KOH	154	68	Catalytic etching (ex-situ)	Powder	[18]
**CoMoC/Ti_3C_2-NC**	1 M KOH	75	43	Liquid etching, annealing (ex-situ)	Powder	[26]
**BCF/Mo_2C-0.4**	1 M KOH	71	52.4	Hydrothermal carbonization (ex-situ)	Self-supported	[27]

**CNTs/$Ti_3C_2T_x$**	1 M KOH	93	128	Liquid etching, annealing (ex-situ)	Powder	[28]
**Ni-GF/VC**	1 M KOH	128	80	Hydrothermal annealing (in-situ)	Self-supported	[29]
**Ni_3C/CNT**	1 M KOH	132	49	ALD (ex-situ)	Self-supported	[30]
**Fe–Ni_3C**	1 M KOH	292	41.3	Precursor method, annealing (ex-situ)	Powder	[31]
**Ni-VC@C**	1 M KOH	146	105	Chemical vapour carbonization reaction (ex-situ)	Powder	[32]
**WC-N/W-1200**	1 M KOH	104	62.2	Atmosphere sintering (in-situ)	Self-supported	This work

Table S9. C and N element content in WC-N/W-1200 electrode obtained by EPMA.

Point	C (mol%)	N (mol%)	C/N
**1**	50.8	4.3	11.8
**2**	51.6	4.6	11.3
**3**	51.4	4.6	11.2
**4**	51.2	4.3	11.8
**5**	52.7	4.1	12.9
**6**	51.6	4.7	10.9
**7**	52.5	4.4	12.0
**8**	50.8	4.9	10.4
**9**	50.4	4.5	11.1
**10**	51.0	4.5	11.5
	**Average value**		11.5

Table S10. Lattice constants of W, WC, WC-N/W and WC/W_2N heterostructure.

Lattice constants	W	WC	WC-N/W	WC/W_2N
a (Å)	3.20795	2.92871	5.99355	5.9501
b (Å)	3.20795	2.92871	5.81150	5.8823
c (Å)	3.20795	2.84987	13.3208	19.1059
α (°)	90	90	90.0084	90
β (°)	90	90	90.0122	90
γ (°)	90	120	90.0016	105

References

[1] X. Fan, H. Zhou, X. Guo, *ACS Nano* **2015**, 9, 5125.

[2] M. Zeng, Y. Chen, J. Li, H. Xue, R. G. Mendes, J. Liu, T. Zhang, M. H. Rümmeli, L. Fu, *Nano Energy* **2017**, 33, 356.

[3] N. Han, K. R. Yang, Z. Lu, Y. Li, W. Xu, T. Gao, Z. Cai, Y. Zhang, V. S. Batista, W. Liu, X. Sun, *Nat. Commun.* **2018**, 9, 924.

[4] Z. Chen, W. Gong, S. Cong, Z. Wang, G. Song, T. Pan, X. Tang, J. Chen, W. Lu, Z. Zhao, *Nano Energy* **2020**, 68, 104335.

[5] L.-N. Zhang, Y.-Y. Ma, Z.-L. Lang, Y.-H. Wang, S. U. Khan, G. Yan, H.-Q. Tan, H.-Y. Zang, Y.-g. Li, *J. Mater. Chem. A* **2018**, 6, 15395.

[6] M. Yao, B. Wang, B. Sun, L. Luo, Y. Chen, J. Wang, N. Wang, S. Komarneni, X. Niu, W. Hu, *Appl.Catal. B: Environ.* **2021**, 280, 119451.

[7] G. Yan, C. Wu, H. Tan, X. Feng, L. Yan, H. Zang, Y. Li, *J. Mater. Chem. A* **2017**, 5, 765.

[8] Q. Gong, Y. Wang, Q. Hu, J. Zhou, R. Feng, P. N. Duchesne, P. Zhang, F. Chen, N. Han, Y. Li, C. Jin, Y. Li, S. T. Lee, *Nat. Commun.* **2016**, 7, 13216.

[9] Z. Kou, T. Wang, H. Wu, L. Zheng, S. Mu, Z. Pan, Z. Lyu, W. Zang, S. J. Pennycook, J. Wang, *Small* **2019**, 15, e1900248.

[10] Y. Gao, Z. Lang, F. Yu, H. Tan, G. Yan, Y. Wang, Y. Ma, Y. Li, *ChemSusChem* **2018**, 11, 1082.

[11] J. Diao, Y. Qiu, S. Liu, W. Wang, K. Chen, H. Li, W. Yuan, Y. Qu, X. Guo, *Adv. Mater.* **2020**, 32, e1905679.

[12] B. Ren, D. Li, Q. Jin, H. Cui, C. Wang, *J. Mater. Chem. A* **2017**, 5, 13196.

[13] L. Huo, B. Liu, Z. Gao, J. Zhang, *J. Mater. Chem. A* **2017**, 5, 18494.

[14] Y. Hu, B. Yu, M. Ramadoss, W. Li, D. Yang, B. Wang, Y. Chen, *ACS Sustain. Chem. Eng.* **2019**, 7, 10016.

[15] Y. Liu, G. D. Li, L. Yuan, L. Ge, H. Ding, D. Wang, X. Zou, *Nanoscale* **2015**, 7, 3130.

[16] Y. Ling, F. M. D. Kazim, Q. Zhang, S. Xiao, M. Li, Z. Yang, *J. Hydrogen Energy* **2021**, 46, 9699.

[17] W. Liu, X. Wang, F. Wang, K. Du, Z. Zhang, Y. Guo, H. Yin, D. Wang, *Nat. Commun.* **2021**, 12, 6776.

[18] H. Yan, Y. Xie, Y. Jiao, A. Wu, C. Tian, X. Zhang, L. Wang, H. Fu, *Adv. Mater.* **2018**, 30, 1704156.

[19] X. Zhang, J. Wang, T. Guo, T. Liu, Z. Wu, L. Cavallo, Z. Cao, D. Wang, *Appl.Catal. B:*

Environ. **2019**, 247, 78.

[20] Z. Yu, K. Mao, Y. Feng, *J. Adv. Ceram.* **2021**, 10, 1338.

[21] R. Kumar, A. Gaur, T. Maruyama, C. Bera, V. Bagchi, *ACS Appl. Mater. Interfaces* **2020**, 12, 57898.

[22] S. Li, P. Tuo, J. Xie, X. Zhang, J. Xu, J. Bao, B. Pan, Y. Xie, *Nano Energy* **2018**, 47, 512.

[23] W. Yuan, L. Cheng, Y. An, H. Wu, N. Yao, X. Fan, X. Guo, *ACS Sustain. Chem. Eng.* **2018**, 6, 8976.

[24] H. Zhang, Z. Ma, G. Liu, L. Shi, J. Tang, H. Pang, K. Wu, T. Takei, J. Zhang, Y. Yamauchi, J. Ye, *NPG Asia Mater.* **2016**, 8, e293.

[25] J. J. Huang, X. Q. Liu, F. F. Meng, L. Q. He, J. X. Wang, J. C. Wu, X. H. Lu, Y. X. Tong, P. P. Fang, J. Electroanal. Chem. 2020, 856, 113727.

[26] X. Wu, S. Zhou, Z. Wang, J. Liu, W. Pei, P. Yang, J. Zhao, J. Qiu, *Adv.* Energy *Mater.* **2019**, 9, 1901333.

[27] J. Xiao, Y. Zhang, Z. Zhang, Q. Lv, F. Jing, K. Chi, S. Wang, *ACS Appl. Mater. Interfaces* **2017**, 9, 22604.

[28] X. Wang, S. Wang, J. Qin, X. Xie, R. Yang, M. Cao, *Inorg. Chem.* **2019**, 58, 16524.

[29] C. Yang, R. Zhao, H. Xiang, J. Wu, W. Zhong, W. Li, Q. Zhang, N. Yang, X. Li, *Adv.* Energy *Mater.* **2020**, 10, 2070152.

[30] W. Xiong, Q. Guo, Z. Guo, H. Li, R. Zhao, Q. Chen, Z. Liu, X. Wang, *J. Mater. Chem. A* **2018**, 6, 4297.

[31] H. Fan, H. Yu, Y. Zhang, Y. Zheng, Y. Luo, Z. Dai, B. Li, Y. Zong, Q. Yan, *Angew. Chem. Int. Ed.* **2017**, 56, 12566.

[32] L. Peng, J. Shen, L. Zhang, Y. Wang, R. Xiang, J. Li, L. Li, Z. Wei, *J. Mater. Chem. A* **2017**, 5, 23028.
